# Immune Checkpoint Inhibitors in Malignant Melanoma: Anti-PD-1, Anti-CTLA-4 and Anti-LAG-3 Therapies

**DOI:** 10.1007/s11912-026-01750-1

**Published:** 2026-03-06

**Authors:** Andrea M. Allen-Tejerina, Periklis Giannakis, Thomas Ho Lai Yau, Christopher R. T. Hillyar, Kathrine S. Rallis

**Affiliations:** 1 Lister Hospital, Stevenage, UK; 2 Barts and The London School of Medicine and Dentistry, Queen Mary University of London, London, UK; 3 Green Templeton College, University of Oxford, Oxford, UK; 4 Barts Cancer Institute, Queen Mary University of London, London, UK

**Keywords:** Cancer biology, Cancer immune surveillance, Immune checkpoint inhibitors, Melanoma, Metastasis.

## Abstract

**Purpose of Review:**

Despite advances over the past decade, malignant melanoma remains associated with poor survival outcomes and an increasing incidence, particularly in older populations. Traditional radio- and chemotherapeutic approaches have shown limited efficacy, whereas immunotherapy has emerged as a promising treatment option owing to the immunogenic nature of most melanoma subtypes. This review aims to explore the biological rationale for immune checkpoint inhibition in melanoma and its therapeutic implications.

**Recent Findings:**

Advances in understanding physiologic immune checkpoint regulation through co-stimulatory and co-inhibitory pathways have led to the development of effective immune checkpoint inhibitors (ICIs), particularly those targeting PD-1 and CTLA-4. These agents have significantly improved overall survival in melanoma; however, a substantial proportion of patients either fail to respond or eventually develop resistance. Ongoing clinical studies are elucidating mechanisms of immune evasion, refining response prediction biomarkers, and exploring combination strategies to overcome resistance and enhance durable remission.

**Summary:**

Immune checkpoint inhibition represents a major therapeutic milestone in malignant melanoma, transforming outcomes for many patients. Nevertheless, resistance and non-responsiveness remain key clinical challenges. Continued investigation into tumor–immune system interactions and rational combination approaches will be critical for optimizing the efficacy and durability of ICIs in melanoma treatment.

## Introduction

Melanoma is a malignant neoplasm of the skin that arises from unrestrained melanocyte proliferation [1]. Although representing only 5% of all skin cancer cases, melanoma accounts for the majority of skin cancer deaths [2, 3]. US statistics from Surveillance, Epidemiology, and End Results (SEER) Program report a 94.1% 5-year relative survival rate for melanoma patients between 2014 and 2020, however, this decreases to 35% when metastatic spread occurs [4]. The incidence of melanoma has increased dramatically over the last 50 years [5–7] surpassing all other cancers in the fair-skinned populations in Europe, North America and Oceania. This rise has been driven by new diagnoses in people over 60 years of age [8]. 

Melanoma is an aggressive malignancy with high metastatic potential and a poor prognosis, the latter due to resistance to cytotoxic chemotherapies [9, 10]. Malignant melanoma demonstrates a high median tumour mutational burden (TMB) – owing to a pathogenesis typically involving ultraviolet (UV)-radiation DNA damage – that initially promotes an immunogenic tumour microenvironment that is associated with higher response rates to immune checkpoint inhibitor (ICI) treatment [11]. However, this can also generate selection pressure favoring immune-evasive mutations, facilitating disease progression and resistance to ICIs. For example, downregulation of MHC class I molecules on melanoma cells reduces antigen presentation to effector immune cells, conferring resistance to ICIs [12]. Impaired antigen presentation can lead to the accumulation of dysfunctional anti-cancer T-cells in the tumor microenvironment, often reinforced by immunosuppressive cancer-associated fibroblasts [13].

Conventional chemotherapy, radiotherapy, and surgical resection are often unable to produce durable remission, lending only to a temporary reduction in tumour burden prior to eventual recurrence. Immunotherapies have revolutionised the treatment landscape for melanoma and are now considered a cornerstone in the management of this disease, particularly in the metastatic and adjuvant settings. This review explores strategies to restore effective anti-cancer immune responses through immune checkpoint inhibition [14], with a focus on anti-PD-1, anti-CTLA-4, and anti-LAG-3 therapies, which are the most extensively studied and currently approved ICIs for melanoma management. It discusses the underlying principles of ICIs, their role in melanoma treatment, the limitations of these therapies, and new avenues for research.

## Review of Literature

### Normal Immune System and the Role of Immune Checkpoints

The immune system protects the host from pathogens, toxins, and mutagenic changes [15]. Responses are classified as innate and adaptive, avoiding host damage by distinguishing self from non-self [16]. T cell receptor (TCR)-mediated antigen recognition is central to adaptive immunity, with antigens presented on MHC class I (normal cells) or class II (APCs).

CD8 T cells recognise MHC class I molecules and act as cytotoxic T lymphocytes (CTLs). CD4⁺ T cells recognize MHC class II and orchestrate responses via cytokine release, differentiating into Th1, Th2, Th17, and Tregs. Tregs maintain self-tolerance. Effector T cells act rapidly, while memory T cells – central memory (T_CM_), effector memory (T_EM_), and tissue-resident memory (T_RM_) cells, although stem cell-like memory (T_SCM_) and effector memory re-expressing CD45RA (T_EMRA_) cells – provide long-term immunity [17].

Other immune cells, including natural killer (NK) cells and γδ T cells, complement T-cell immunity. NK cells target cells with downregulated MHC class I, while γδ T cells bridge innate and adaptive immunity, recognising antigens independent of MHC molecules. Together, these components establish a robust, regulated defence system. Central and peripheral tolerance prevent inappropriate activation by self-antigens [18].

#### Central Tolerance

During T-cell maturation, V(D)J recombination generates diverse TCRs. Central tolerance in the thymus eliminates self-reactive T-cells via negative selection under AIRE regulation [19]. Positive selection ensures T-cells capable of interacting with MHC molecules survive. T-cells with non-interacting TCRs are deleted [16, 18].

#### Peripheral Tolerance

Peripheral tolerance maintains T-cell quiescence. Naïve T-cells encountering antigen undergo activation proportional to affinity and density [20]. Mechanisms preventing self-reactive T-cell activation include quiescence, ignorance, anergy, exhaustion, senescence, and apoptosis [21]. Ignorance occurs when antigen density is low or localized. Chronic antigen exposure induces exhaustion, a reversible dysfunction with reduced cytokine production and effector function, or senescence, a permanent cessation of proliferation and activity [21].

#### Two-signal Hypothesis for T-cell Activation

**Fig. 1:**
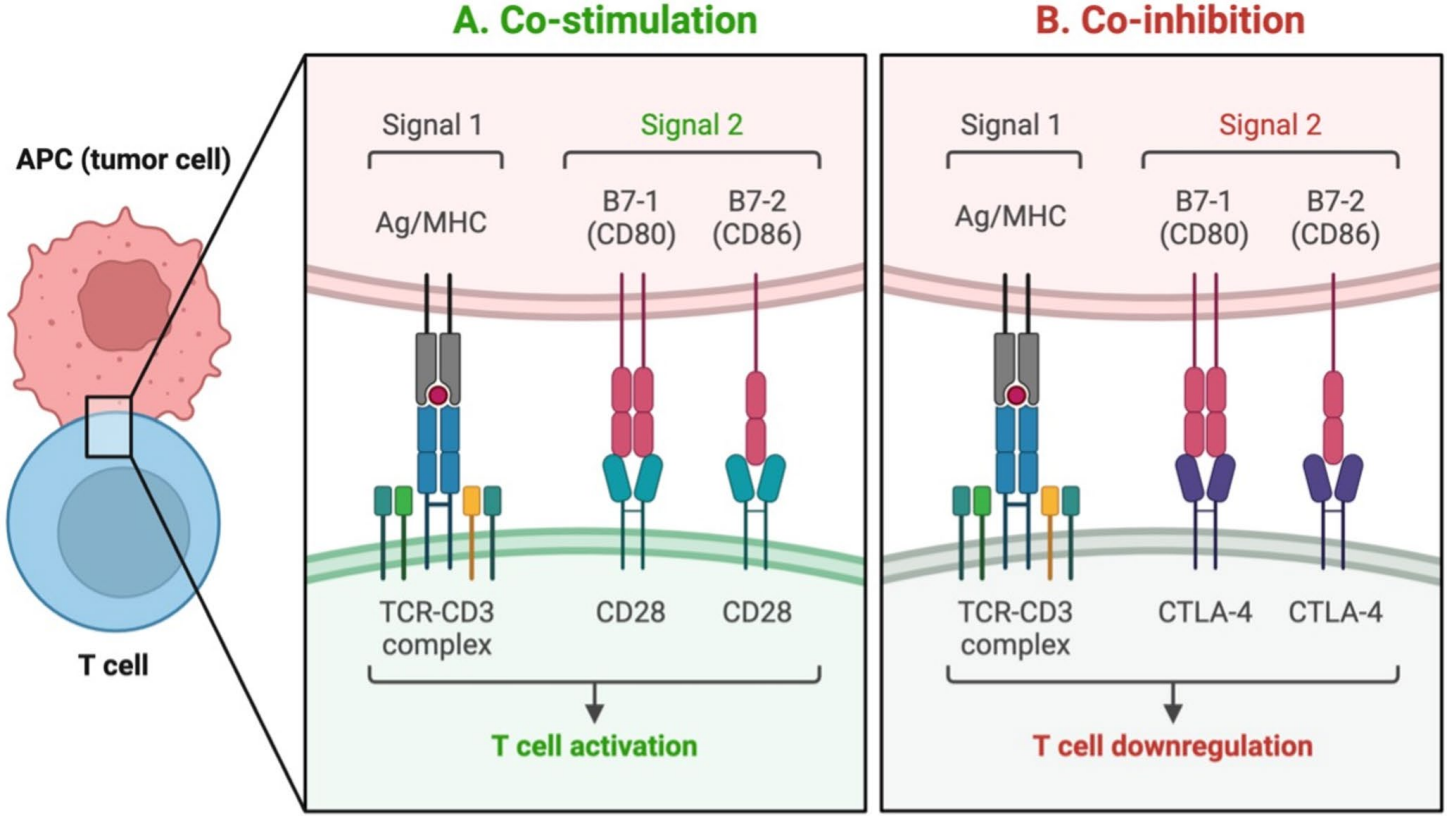
Two-signal hypothesis for T-cell activation (**A**) vs. co-inhibitory signal (**B**). **A** Two signals are required for T-cell activation. Signal 1 is produced from the interaction of TCR on T-cells with the Ag/MHC ligand on APC. Signal 2 is produced from the interaction of co-stimulatory receptors such as CD80 and CD86 on the APC with co-receptors including CD28 on T-cells. In the presence of only signal 1, T-cells undergo tolerance, anergy or apoptosis, whereas in the presence of both signal 1 & 2, T-cells undergo activation, clonal expansion and effector functioning. **B** Co-inhibitory receptors, such as CTLA-4, on T-cells prevent the binding of co-stimulatory ligands CD80/86 on APC with CD28 on T-cells as the former exhibit higher avidity and affinity and outcompete CD28. APC, antigen presenting cell; Ag/MHC, antigen bound to major histocompatibility complex; TCR, T-cell receptor; CTLA-4, cytotoxic T-lymphocyte-associated protein 4; CD, cluster domain. Adapted with permission from Rallis et al. [24]

**Fig. 2:**
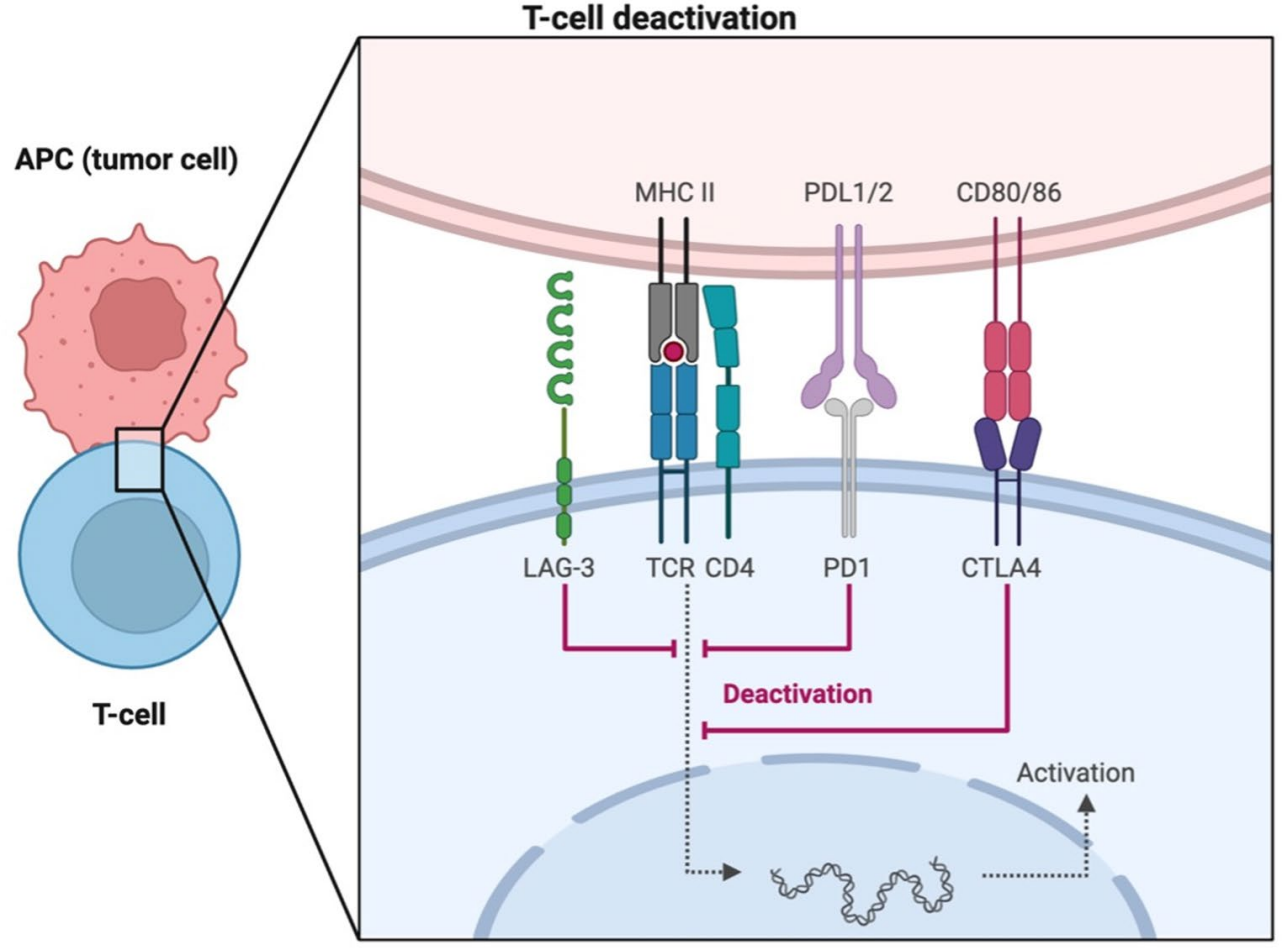
Co- inhibitory signals. APC, antigen presenting cell; MHC-II, major histocompatibility complex class 2; TCR, T-cell receptor; CTLA-4, cytotoxic T-lymphocyte-associated protein 4; PD-1, programmed cell death protein 1; PD-L1/2, programmed death-ligand 1 and 2; CD, cluster domain; LAG-3, lymphocyte activate gene-3. Adapted from Mariuzza et al. [25]

T-cell activation requires two signals: TCR recognition of antigen-MHC and CD28 binding to CD80/CD86 (Fig. 1), triggering intracellular cascades and cytokine production [22]. Co-inhibitory signals – including CTLA-4, PD-1, LAG-3, and TIGIT – oppose activation to prevent dysregulated responses (Fig. 2) [23].

#### Co-stimulatory Signals

CD28 is constitutively expressed on naïve CD4⁺ (~ 95%) and CD8⁺ (~ 50%) T-cells. It is a homodimeric 44-kDa glycoprotein with extracellular, transmembrane, and intracytoplasmic domains. CD28 ligation prevents T-cell anergy via BCL-xL upregulation, protecting against CD95-mediated apoptosis [26, 27]. Interactions with CD80/CD86 are enhanced by TCR stimulation, while overstimulation induces negative feedback. CTLA-4 expression upregulated upon sustained CD28-dependent signaling exerts inhibitory effects on T-cells [22, 28–30].

#### Co-inhibitory Signals

**Fig. 3:**
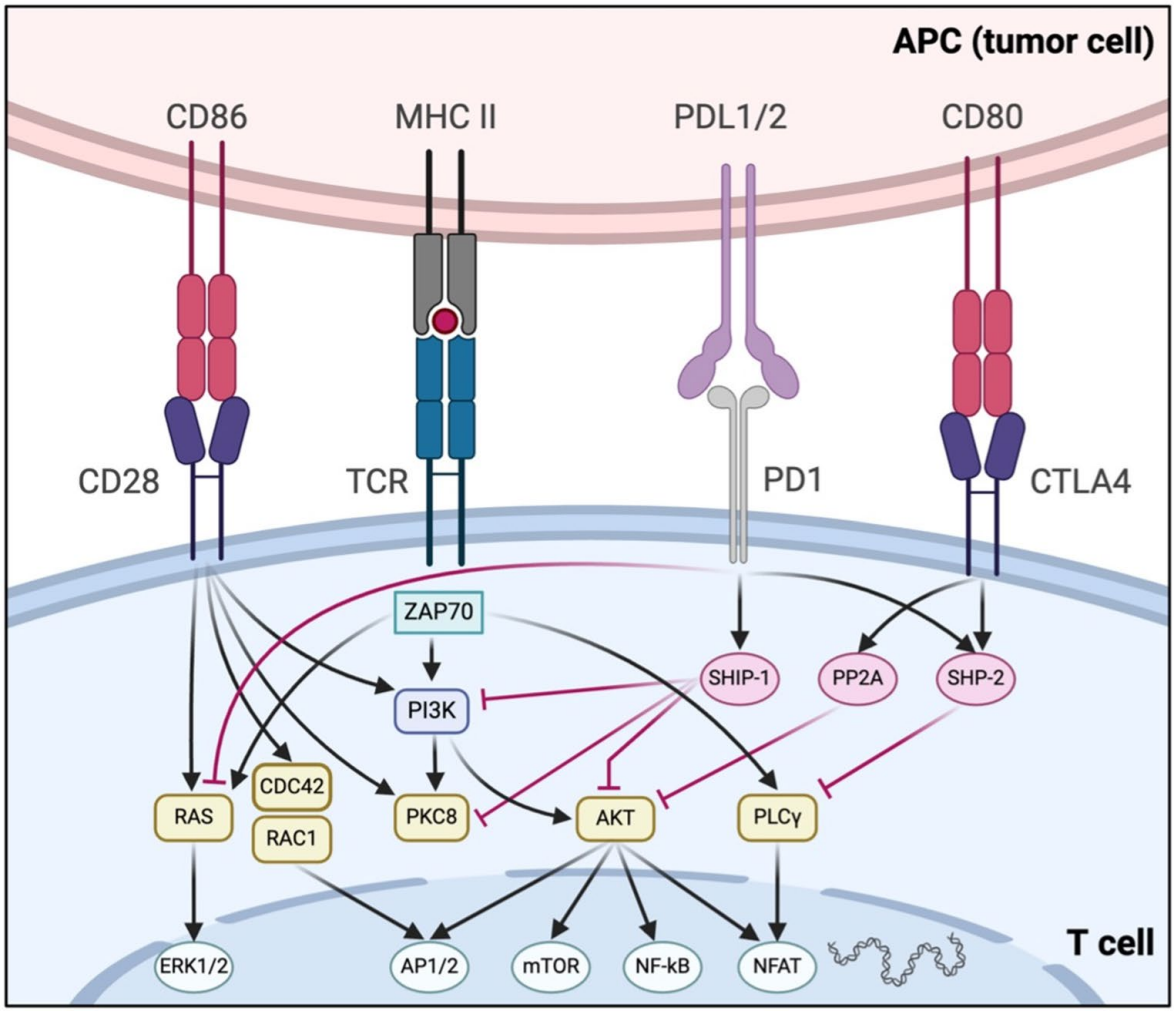
Interaction of B7/CD28 family of receptors including co-inhibitory receptor systems CTLA-4 and PD-1 and their downstream signalling targets. Black arrows, positive co-stimulatory signals; red lines, negative co-inhibitory signals; APC, antigen presenting cell; MHC, major histocompatibility complex; TCR, T-cell receptor; CTLA-4, cytotoxic T-lymphocyte-associated protein 4; PD-1, programmed cell death protein 1; PD-L1/2, programmed death-ligand 1 and 2; CD, cluster of differentiation; ZAP, zeta-chain-associated protein kinase; PI3K, phosphatidylinositol-3-kinase; PKC, protein kinase C; AKT, serine/threonine protein kinase also known as protein kinase B (PKB); PLC, phospholipase C; SHP1/2, Src homology 2 domain tyrosine phosphatase 1; PP2A, serine/threonine protein phosphatase 2 A; RAS, rat sarcoma protein; RAC1, Ras-related C3 botulinum toxin substrate 1 protein; CDC42, cell division control protein 42 homolog; ERK, extracellular signal-regulated kinase; AP1/2, activator protein 1 and 2; mTOR, mammalian target of rapamycin; NF-κB, nuclear factor kappa-light-chain-enhancer of activated B cells; NFAT, nuclear factor of activated T-cells. Adapted with permission from Rallis et al. [24]

**Fig. 4:**
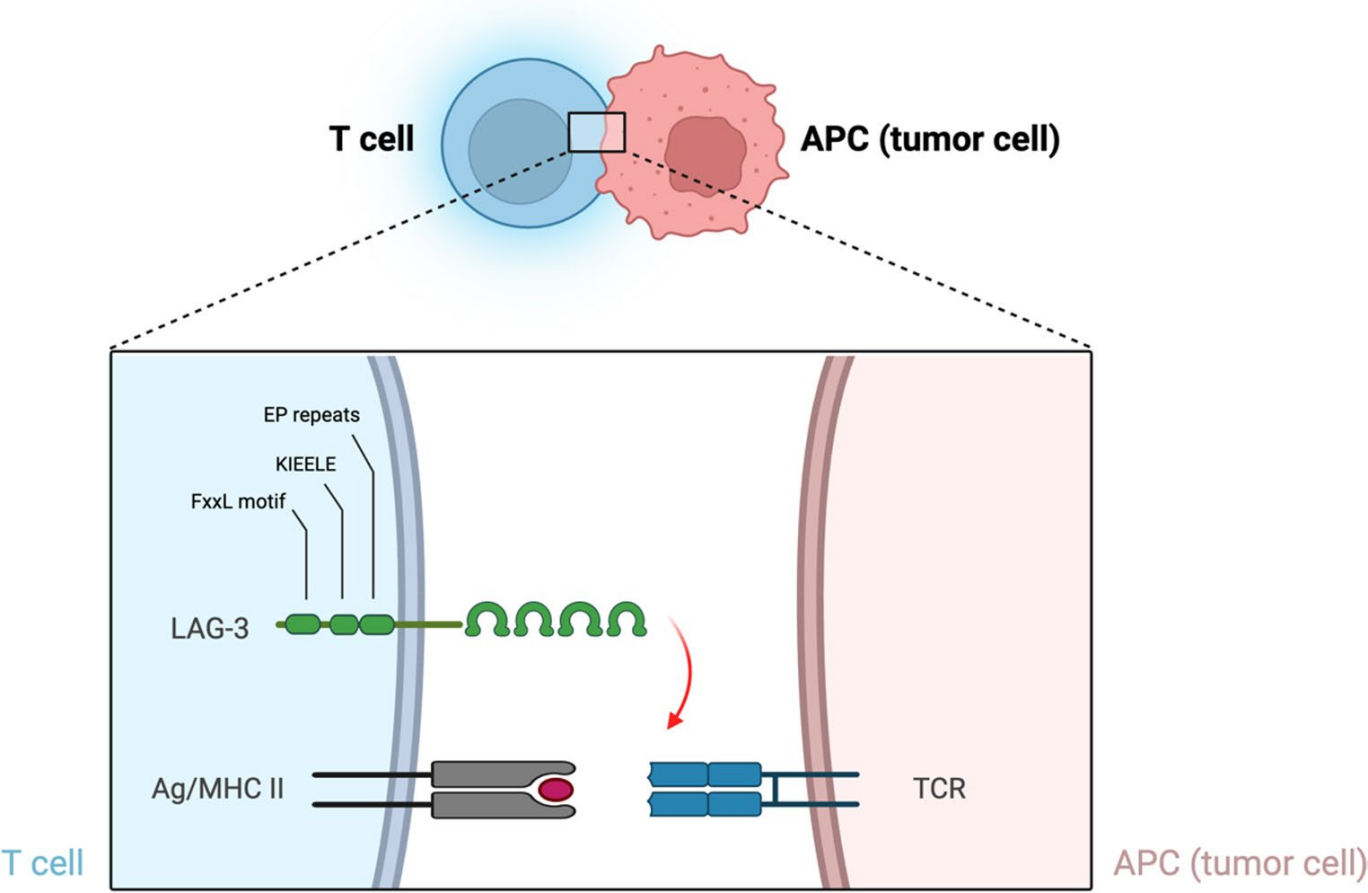
LAG-3 mechanism of action. Red arrow, negative co-inhibitory signal; APC, antigen presenting cell; MHC, major histocompatibility complex; TCR, T-cell receptor; LAG-3, lymphocyte activate gene-3; EP, glutamic acid-proline. Adapted from Mariuzza et al. [25]

CTLA-4, a CD28 homolog, is transiently expressed on CD4⁺/CD8⁺ T-cells and constitutively on Tregs [31, 32]. It competes with CD28 for B7 ligands and recruits phosphatases to inhibit TCR/CD28 signaling [33–35]. Further, CTLA-4 actively inhibits T-cell co-stimulation by recruiting intracellular phosphatases of the Src homology 2 (SH2) domain family, including tyrosine phosphatase 1 (SHP1), SHP2, and serine/threonine protein phosphatase 2 A (PP2A) (Fig. 3) [35, 36]. PD-1 regulates peripheral responses via PD-L1/PD-L2 and inhibits key signaling pathways [37–39]. LAG-3 interacts with MHC-II and other ligands, suppressing T-cell proliferation and cytokine production; co-expressed with PD-1 to synergistically attenuate immune responses (Fig. 4) [39–47].

### The Immune System in Cancer

Cancer cells generate neoantigens capable of eliciting immune responses [31, 35, 48]. Tumors evade immunity via (a) antigen/MHC downregulation, (b) secretion of immunosuppressive factors and recruitment of Tregs/Bregs/MDSCs/fibroblasts, and (c) upregulation of immune checkpoints like PD-1, CTLA-4, LAG-3, and IDO [14, 49, 50].

#### Immune Exhaustion

T-cell exhaustion, described in chronic viral infection, also occurs in melanoma, with impaired proliferation, cytotoxicity, cytokine secretion, mitochondrial function, and upregulation of inhibitory receptors (CTLA-4, PD-1, LAG-3, TIGIT, TIM-3) [14, 31]. Co-expression of multiple inhibitory receptors correlates with severe dysfunction and absence of CD4⁺ T-cell support [14].

### Immune Checkpoint Inhibitors for Melanoma Therapy

Immune checkpoint blockade aims to prevent the downstream signalling mediated by CTLA-4, PD-1 and LAG-3. This, in turn, avoiding the induction of anergy and restraint of the immune response against tumour cells. ICIs that have been approved by the FDA for treatment of melanoma include ipilimumab (anti-CTLA-4; Yervoy^®^), pembrolizumab (anti-PD-1; Keytruda^®^), nivolumab (anti-PD-1; Opdivo^®^), combination nivolumab-ipilimumab (anti-PD-1, anti-CTLA-4; Yervoy^®^) and combination nivolumab-relatlimab (anti-PD-1, anti-LAG-3; Opdualag^®^) [51–53]. ICIs are approved in the adjuvant setting (following resection of high-risk primaries) and in the metastatic setting.

#### Rationale for Initial Immunotherapy Breakthroughs in Melanoma

Historically, treatment options for malignant melanoma were limited, with conventional therapies such as surgery, radiotherapy, and chemotherapy offering only modest survival benefits. Dacarbazine, the first chemotherapeutic agent approved for metastatic melanoma in 1975, remained the standard of care for decades, but its efficacy was poor, with response rates of only 10% to 15%, a median survival time of six months, and a one-year overall survival rate of 25% [54].

The need for more effective treatments led to the exploration of immunotherapy, especially given melanoma’s high TMB, which contributes to its immunogenicity. The large number of DNA mutations in melanoma cells, largely induced by UV radiation, results in altered protein sequences that are processed and displayed as tumour-specific neoantigens on MHC molecules. These neoantigens are recognized by host T-cells as foreign, making melanoma an ideal candidate for immunotherapy [55]. The recognition of melanoma’s immunogenic potential spurred early immunotherapy efforts, with high-dose interleukin-2 (IL-2) becoming one of the first FDA-approved treatments for melanoma in 1998. IL-2 therapy facilitated the expansion of T-cells capable of targeting melanocyte differentiation antigens like gp100 and MelanA [55, 56]. Although the response rates were relatively low—around 16%—and the complete response rate was only 6% [57], the ability to generate durable immune responses against cancer cells paved the way for further development of immune-based treatments in melanoma and other malignancies.

#### Clinical Efficacy of Checkpoint Inhibitors Leading Market Authorisation in Melanoma

I) Ipilimumab

Ipilimumab is currently approved for use in advanced melanoma, as an adjuvant in resected stage III melanoma and as combination with nivolumab. Ipilimumab was the first ICI approved in 2011 for the treatment of unresectable or metastatic melanoma [53]. This approval was based on the results from the pivotal MDX010-020 trial (NCT00094653) in unresectable, metastatic melanoma. This phase 3 trial randomised patients in a 3:1:1 ratio to receive either [1] ipilimumab (3 mg/kg) plus the gp100 peptide vaccine [2], ipilimumab (3 mg/kg) plus placebo, or [3] the gp100 vaccine alone. Results demonstrated that median OS was significantly improved with ipilimumab, either with or without the gp100 vaccine, compared to the gp100 vaccine alone. Median OS was 10.0 months in the ipilimumab plus gp100 group, 10.1 months in the ipilimumab monotherapy group, and 6.4 months in the gp100 vaccine alone group (hazard ratio [HR] for OS: 0.68; 95% CI: 0.55–0.85, *p* < 0.001) [58].

On October 18, 2015, the FDA approved ipilimumab as an adjuvant treatment in patients with cutaneous melanoma based on the randomised CA 184 − 029 study [59]. Its approval was further expanded to include paediatric patients aged 12 years or older on July 24, 2017, following an open-label, single-arm trial. The approved dosing regimen consisted of 3 mg/kg administered intravenously every three weeks for a total of four doses [53].

II) Pembrolizumab

Following the approval of ipilimumab, the anti-PD-1 ICIs nivolumab and pembrolizumab also received approval for the treatment of melanoma and other malignancies. Pembrolizumab is currently approved for use in advanced melanoma and as an adjuvant in resected melanoma. Pembrolizumab received approval in 2014 for the treatment of unresectable or metastatic melanoma in patients previously treated with CTLA-4 or BRAF inhibitors based on the results of the KEYNOTE-001 trial (NCT01295827) [60]. This phase 1b study demonstrated the durable antitumour activity and tolerability of pembrolizumab after 5-year follow-up data for patients with advanced melanoma. The 5-year survival rate for patients receiving pembrolizumab was 34% (73% with ongoing response at year 5) in all patients and 41% (82% with ongoing response) in treatment-naïve patients [61]. 

KEYNOTE-006, a phase III clinical trial comparing pembrolizumab to ipilimumab in 834 adult patients with stage 3/4 BRAF V600 positive melanoma that had received no more than one prior systemic therapy, demonstrated a significant increase in 6-month progression free survival (PFS) (47.3% vs. 26.5%) and 12-month OS (74.1% vs. 58.2%) [62]. Response rates were higher with pembrolizumab (33.7% vs. 11.9%), and grade 3–5 adverse events were reduced (13.3% vs. 19.9%) in comparison to ipilimumab [62]. This trial led to the FDA approval of pembrolizumab as a first-line treatment for advanced melanoma, regardless of BRAF mutation [53].

The FDA extended the use of pembrolizumab in the adjuvant setting in patients with resected stage III melanoma based on the KEYNOTE-054 (EORTC 1325-MG) trial. This phase 3 randomised, double-blind study demonstrated prolonged RFS in patients treated with pembrolizumab compared to placebo (1-year rate of RFS 75.4% [95% CI, 71.3 to 78.9] vs. 61.0% [95% CI, 56.5 to 65.1] [63].

III) Nivolumab

By comparison, nivolumab, exhibits similar efficacy to pembrolizumab. Nivolumab is currently approved for advanced melanoma, in the adjuvant setting and in combination with ipilimumab. Nivolumab was first approved in 2014 for the treatment of unresectable or metastatic melanoma [64]. This approval was based on the results from the CheckMate-037 trial (NCT01721746), a phase 3 study comparing nivolumab to chemotherapy (either dacarbazine or paclitaxel) in patients with advanced melanoma who had previously received ipilimumab and, if appropriate, a BRAF inhibitor. Results demonstrated that nivolumab significantly improved OS and PFS compared to chemotherapy. The median OS was not reached in the nivolumab group versus 10.8 months in the chemotherapy group (hazard ratio [HR] for OS: 0.42; 95% CI: 0.31–0.58, *p* < 0.001). The ORR was 31.7% in the nivolumab group compared to 10.6% in the chemotherapy group (*p* < 0.001) [65]. 

Nivolumab was later approved as an adjuvant treatment for melanoma based on the CheckMate-238 trial (NCT02388906), a phase 3 study comparing nivolumab to ipilimumab in patients with resected, high-risk stage III or IV melanoma. The trial demonstrated that nivolumab significantly reduced the risk of recurrence or death compared to ipilimumab, with a 3-year recurrence-free survival (RFS) rate of 66.4% in the nivolumab group versus 52.0% in the ipilimumab group (hazard ratio [HR] for recurrence or death: 0.65; 95% CI: 0.51–0.83, *p* < 0.001) [66].

IV) Combination nivolumab and ipilimumab

To explore OS benefit, a phase I study showed that ipilimumab plus nivolumab conferred a 63% 3-year OS in 94 advanced melanoma patients [67]. This was followed by two landmark clinical trials: CHECKMATE-067 and CHECKMATE-069. These lead to the FDA approval of ipilimumab plus nivolumab combination therapy for unresectable or metastatic melanoma regardless of BRAF status. The phase 2 trial CHECKMATE-069 compared the clinical outcomes between ipilimumab plus nivolumab to ipilimumab alone and found an increased 2-year OS (53.6% to 63.8%) compared to ipilimumab alone [68]. The phase 3 CHECKMATE-067 trial found that OS at 3 years increased to 58% when using dual therapy compared to 52% of the nivolumab and 34% of ipilimumab monotherapy groups [69]. Hence, in 2015, nivolumab plus ipilimumab received accelerated FDA approval for BRAF V600 wild-type, unresectable or metastatic melanoma. In 2016, approval was expanded to include unresectable or metastatic melanoma with or without BRAF mutations. Currently, dual ICI with ipilimumab plus nivolumab is the most effective treatment for advanced melanoma and therefore considered first line, yet it is also the most toxic treatment for advanced melanoma, leading to approximately 40% of patients to discontinue treatment prematurely [70]. Indeed, dual ICI regimens have been questioned around the increased risk of irAEs and limited survival benefits in some patient subgroups. A meta-analysis of nine RCTs demonstrated that the incidence of adverse effects (described by 8 included studies) was overall significantly higher with combination ICI compared to monotherapy with either nivolumab or ipilumumab (RR 1.07, 95% CI [1.03–1.12] with a p-value = < 0.001) [71].

Low-dose ICI combinations are being investigated to reduce toxicity and enhance efficacy [72]. Two clinical trials have demonstrated significant antitumour activity, safety, and comparable tolerability from low-dose dual ICI therapy with anti-PD-(L)1 plus ipilimumab in melanoma patients who progressed on prior anti-PD-(L)1 monotherapy [73, 74]. SWOG S1616, a randomized phase II trial, evaluated ipilimumab with or without nivolumab in PD-1/PD-L1–refractory metastatic melanoma and found that the combination improved response rates (28% vs. 9%) and PFS compared with ipilimumab alone, supporting its use as a second-line strategy in PD-1-resistant disease [75]. These findings support dual ICI therapy as second-line treatment for melanoma that progresses on anti-PD-(L)1 monotherapy [53, 72].

V) Combination nivolumab and ipilimumab

Relatlimab, a LAG-3 blocking antibody, is the fourth approved ICI for the treatment of patients with unresectable or metastatic melanoma [70]. The combination of relatlimab plus nivolumab was explored in RELATIVITY-047, a phase 2/3 randomised double-blind trial which demonstrated increased efficacy and safety compared to nivolumab monotherapy. The RELATIVITY-047 trial led to the approval of relatlimab in combination with nivolumab in March 2022 by the FDA and in September 2022 by the European Medicines Agency (EMA). Combination relatlimab plus nivolumab has shown a risk of treatment-related adverse effects of 21% compared to 59% when using ipilimumab plus nivolumab [65]. Prior to this, the RELATIVITY-020 study (NCT01968109), encompassing patients with advanced melanoma who had not responded or progressed on anti-PD-1 therapy, demonstrated promising tolerability and sustained response to the combination of relatlimab and nivolumab. The study reported an overall response rate (ORR) of 16% and a disease control rate of 45% [76]. In the future, should the survival advantage of relatlimab-nivolumab match or surpass that of nivolumab-ipilimumab, combination therapy with relatlimab-nivolumab may emerge as the fresh standard of care for advanced melanoma in previously untreated patients [77].

Other combinations involving LAG-3 are currently being explored in phase 3 clinical trials for melanoma. These trials are evaluating the combination of fianlimab (anti-LAG-3) and cemiplimab (anti-PD-1) versus pembrolizumab in patients with previously untreated unresectable melanoma (NCT05352672) and in patients with completely resected high-risk melanoma (NCT05608291) [78].

### Immune Checkpoint Inhibitors as Neoadjuvant Therapy

The rationale for utilising ICIs in the neoadjuvant setting for melanoma lies in the concept that administration of ICIs while the primary tumour is still present will result in a more robust T cell response, due to a higher tumour burden and increased antigen presentation from tumour cells. Both preclinical and clinical trials have shown that patients treated in the neoadjuvant setting experience better event-free survival (EFS) outcomes compared to those treated in the adjuvant setting [79].

The phase 2 SWOG S1801 trial showed that neoadjuvant pembrolizumab, followed by adjuvant pembrolizumab, significantly improved EFS compared to adjuvant therapy alone (72% vs. 49% at two years, *p* = 0.004) [80]. New recommendations from ASCO include the use of pembrolizumab for patients with resectable stage IIIB to IV cutaneous melanoma followed by resection and adjuvant pembrolizumab [81]. However, strong evidence not only supports neoadjuvant single-agent anti–PD-1 therapy, but also combination therapy with ipilimumab and nivolumab, and nivolumab and relatlimab [79].

In the phase 2 SWOG S1801 trial, administering three neoadjuvant cycles of pembrolizumab followed by 15 adjuvant cycles resulted in a superior EFS compared to 18 cycles of adjuvant pembrolizumab, with estimated two-year EFS rates of 72% versus 49% (*p* = 0.004). Rates of treatment-related adverse events were comparable between both treatment groups [80]. Other studies, such as the OpACIN and OpACIN-neo trials, have further demonstrated that neoadjuvant combination therapies, including ipilimumab/nivolumab, can induce high pathological response rates and expand tumor-specific T cell clones, potentially reducing relapse rates [82]. However, side effects were considerably increased with the neoadjuvant regime. The OpACIN-neo trial addressed the increased toxicity by reducing the dose of ipilimumab, showing a decrease in adverse effects with ipilimumab at 1 mg/kg and nivolumab at 3 mg/kg [82]. More recently, the phase III NADINA trial provided confirmatory evidence that neoadjuvant nivolumab plus ipilimumab followed by surgery and response-adapted adjuvant therapy significantly improved event-free survival compared with standard adjuvant nivolumab alone, establishing neoadjuvant ICI as a potential new standard of care in resectable stage III melanoma [83].

Despite these promising findings, ICIs have not yet received formal regulatory approved for neoadjuvant use in melanoma in many countries, though it should be noted that neoadjuvant ICI therapy is now incorporated as a standard approach in major clinical guidelines, such as the NCCN. One key reason is the need for more definitive, long-term data on survival outcomes and safety. Early trials indicate that neoadjuvant therapy can lead to increased toxicity, particularly with combination regimens, which may delay surgery or increase perioperative risks [79]. While dose adjustments, as seen in OpACIN-neo, have helped mitigate some toxicity concerns, regulators require further evidence to determine optimal patient selection, treatment duration, and risk-benefit profiles. In contrast, neoadjuvant ICIs have already been approved for non-small cell lung cancer (NSCLC), where studies like CheckMate 816 demonstrated clear survival benefits with manageable toxicity [84]. The approval of ipilimumab and nivolumab in NSCLC highlights the potential for similar strategies in melanoma, but further validation in larger phase 3 trials is needed before neoadjuvant ICIs can become the standard of care in melanoma treatment.

### Combination Chemoradiotherapy with Immune Checkpoint Inhibitors

Chemoradiotherapy (CRT) is standard therapy across many cancers as it offers a survival benefit and increased local disease control rates [85]. Both chemotherapy and radiotherapy have been shown to impact the efficacy of immunotherapies and vice versa. Local tumour irradiation increases MHC-I expression as well as tumour neoantigen presentation and retrieval, helping to facilitate immune-mediated cancer destruction [86]. Some evidence suggests that radiotherapy may also promote innate and adaptive immune responses within the tumour microenvironment in certain settings [87]. The abscopal effect, which describes the regression of cancer sites distal to the primary site of irradiation, has been attributed to immune activation whereby circulating immune cells demonstrate increased responses to neo-antigens following radiotherapy [88]. According to some studies, chemotherapy may also improve tumour neoantigen presentation, sensitise tumours to immunotherapy and, paradoxically, inhibit immunosuppression in certain settings [reviewed in [89].

In melanoma, much of the literature focuses on efficacy and adverse effects [57, 75–78]. Research looking at survival and response rates provides a mixed picture.

I) Chemotherapy and Immune Checkpoint Inhibitors

In many solid tumours, such as lung and breast cancer, the combination of chemotherapy with ICIs has become standard, leveraging chemotherapy’s cytotoxic and immunomodulatory effects to enhance ICI efficacy. In melanoma, this paradigm has also been explored. Robert et al. [90] looked at OS in patients receiving ipilimumab plus dacarbazine, a chemotherapy agent approved by the FDA for advanced melanoma [91] or dacarbazine alone. Results showed that patients receiving a combination of chemotherapy and ipilimumab had an increased OS at 1 year (47.3% relative to 36.3% in patients received chemotherapy alone). The OS continued to be better at 3 years (20.8% compared to 12.2%). However, grade 3 or 4 adverse events were seen in 56.3% of patients treated with ipilimumab and dacarbazine compared to 27.5% in those treated with dacarbazine only.

A randomised Phase I study showed that ipilimumab could be safely combined with dacarbazine or paclitaxel/carboplatin in patients with previously untreated advanced melanoma. However, estimates of ORR were imprecise due to the small size of the study, demonstrating that combination of ipilimumab plus paclitaxel/carboplatin (ORR 27.8%) did not lead to better outcomes compared with ipilimumab alone or ipilimumab plus dacarbazine (ORR 33.3% in both groups) [92].

Importantly, emerging data suggest that the chemo-ICI combination in melanoma may exert activity even after progression on anti-PD-1 therapy. For instance, Aguilera et al. report that in metastatic melanoma patients who had failed PD-1 blockade, adding chemotherapy increased CX3CR1⁺ therapy-responsive CD8⁺ T-cells and was associated with improved clinical responses [93]. Similarly, Goodman et al. describe a case series of three patients treated with the combination of nivolumab plus temozolomide after multiple prior therapies, demonstrating response in the heavily pre-treated setting [94].

Collectively, these findings highlight that while chemo-ICI combinations in melanoma remain less established than in lung or breast cancer, they warrant further investigation – particularly in the post-PD-1 setting or for patients with limited options.

II) Radiotherapy and Immune Checkpoint Inhibitors

Koller et al. (2017) found improved OS in patients with advanced melanoma treated with concurrent ipilimumab and radiotherapy compared to ipilimumab alone (19 months vs. 10 months, *p* = 0.01) [95]. However, a retrospective cohort study of 835 patients who received CTLA-4 or anti-PD-1 with or without preceding radiotherapy for unresectable metastatic melanoma found no survival differences [for anti-CTLA-4 (OS, HR = 1.08, 95% CI = 0.81 to 1.44, *p* = 0.61) and for anti-PD-1 (OS, HR = 0.73, 95% CI = 0.43 to 1.25, *p* = 0.26)] [96].

The outcomes from combining chemotherapy or radiotherapy and immunotherapy for melanoma are diverse, highlighting the necessity for more hypotheses driven research based on a sound biological basis to ascertain both its effectiveness and safety.

## Limitations of Checkpoint Inhibitors

### Treatment Resistance

Treatment resistance is a main limitation of ICIs. Primary resistance or intrinsic resistance occurs when malignant cells do not respond to immunotherapy at initial drug exposure and acquired resistance to ICIs arises from the successive acquisition of immune evasion mechanisms [14]. Primary resistance varies between ICIs. In metastatic melanoma, the ORR with ipilimumab is 15%, PD-1 inhibitors display 30–35% and combination of nivolumab/ipilimumab and nivolumab/relatlimab ~ 60% and 40% respectively. Acquired resistance occurs in 25% within the first two years of ICI [97].

I) Primary Resistance

Primary resistance was experienced by 40–65% of patients receiving PD-1 inhibitors and 70% of patients receiving CTLA-4 inhibitors. ICIs reverse inhibitory signals towards effector T cells, thus all mechanisms that dampen T cell responses cause ICIs to be less effective. These mechanisms include low TMB, impaired dendritic cell maturation leading to impaired antigen presentation and priming, VEGF and ANG2 overexpression, and tumour-infiltrating lymphocytes (TILs) inhibition by Treg cells [98]. Many strategies that target these mechanisms are being explored and consist of combination of radiotherapy and ICI, combination anti-VEGF and ICI, combination anti-VEGF and anti-ANG2, CXC3 upregulation, Treg suppression with sunitinib and cytotoxic T cell therapy [86].

II) Acquired Resistance

Mechanisms that underlie acquired resistance overlap with those of primary resistance. These mechanisms include deficient antigen presentation, loss of β2- microglobulin associated with loss of MHC I expression, dysfunction of JAK1, 2/STAT signalling as well as PD-L1, LAG-3, TIM-3 and FCRL6 overexpression leading to T cell exhaustion. Since treatment with ICI can lead to the upregulation of other inhibitors receptors, developing antagonistic monoclonal antibodies of inhibitory receptors associated with resistance such as TIM-3 aim to overcome resistance. Indeed, combination ICI with dual TIM-3 and PD-1 inhibition has been shown to be more successful in achieving antitumour responses than TIM-3 blockade alone [99]. These findings underly the rationale behind combination of ICI to overcome acquired resistance.

### Toxicity

ICIs face limitations due to immune-related adverse events (irAEs), which result from the activation of self-targeting T cells that harm host tissues [100]. These events often prompt treatment interruptions, delays and in rare cases, pose life-threatening risks. IrAEs resemble autoimmune diseases and are more common in individuals with a predisposition to autoimmunity, high body mass index, chronic smoking, and differ in incidence based on sex – women receiving CTLA-4 inhibitors and men receiving PD-1/PD-L1/ inhibitors appear to be at higher risk [101]. While extensive data exist on CTLA-4 and PD-1/PD-L1-related irAEs, information on LAG-3 inhibitors remains limited.

I) Organ Toxicity

ICIs can affect any organ, but irAEs most commonly involve the colon, liver, lungs, pituitary, thyroid and skin. Though rarer, severe complications affecting the heart, nervous system and other organs can occur [102]. The type and severity of toxicity depend on the class of ICI used:

- CTLA-4 inhibitors tend to prompt gut inflammation and hypophysitis
- PD-1 inhibitors are often associated with thyroiditis, pneumonitis, and autoimmune diabetes
- Combination ICI therapy increases toxicity risk, particularly with dual CTLA-4/PD-1 blockade, which induces more severe irAEs [89].
- LAG-3 inhibitors appear to be better tolerated, with common side effects including rash, fatigue and hypothyroidism [78].

Higher-grade irAEs occur in 30–55% of patients receiving combination ipilimumab/nivolumab, compared to 10–15% with PD-1/PD-L1 monotherapy. Notably, CTLA-4-related toxicities are dose-dependent, whereas PD-1/PD-L1 toxicities are not [103].

II) Toxicity Incidence across Immune Checkpoint Inhibitors

The incidence of severe irAEs tends to follow predictable patterns across large populations:

- **CTLA-4 inhibitors: **20–30% severe irAEs (dose-dependent).
- **PD-1/PD-L1 inhibitors: **15–20% severe irAEs (not dose-dependent).
- **Combination CTLA-4/PD-1 therapy: **40–50% severe irAEs.
- Fatal irAEs: ~0.4% for PD-1/PD-L1 monotherapy; ~1.2% for combination therapy [104].

In contrast, chemotherapy leads to predictable, dose-dependent toxicities like myelosuppression and nausea, with a lower rate of severe side effects (10–20%), that are generally reversible. High-dose IL-2 therapy, once the mainstay of melanoma treatent, carries the highest toxicity burden (60–80% severe toxicity), with side effects ranging from vascular leak syndrome to hypotension, and multi-organ dysfunction requiring ICU-level care [57, 105, 106].

Combination ICI has been shown to carry higher rates of toxicities compared to monotherapy regardless of age [107]. In the CheckMate 067 trial (NCT01844505**)**, approximately 40% of patients discontinued nivolumab/ipilimumab treatment due to toxicity, a notably higher percentage compared to 14% for nivolumab alone and 15% for ipilimumab alone. Overall, 59% of patients receiving the combination therapy encountered grade 3–4 adverse events, a substantially higher rate compared to the 23% observed with nivolumab alone [108]. The risk of toxicity appears to be additive rather than synergistic [109].

LAG-3 inhibitors offer a more favorable safety profile. The RELATIVITY-047 trial (NCT03470922) demonstrated that combining relatlimab with nivolumab resulted in a 21% risk of treatment-related adverse effects, notably lower than the 59% risk when using ipilimumab plus nivolumab. Grade 3–4 adverse events occurred in 18.9% of patients with relatlimab/nivolumab versus 9.7% with nivolumab alone, suggesting manageable toxicity without unexpected safety concerns [110]. Overall, adverse effects related to treatment were seen in 81.1% of patients receiving relatlimab-nivolumab, a rate lower than that observed in the ipilimumab group (86.2%) and the ipilimumab-nivolumab group (95.5%) [111].

III) Timeline

The timing of irAEs varies with the majority appearing in the first 8 weeks of treatment. Cutaneous irAEs tend to develop earlier, followed by gastrointestinal, hepatic, pulmonary, endocrine and renal. Most of these resolve within months, but endocrine irAEs can require long life treatment. Timing of irAEs also varies depending on the ICI. Ipilimumab shows higher rate of side effects in the first 12 weeks, whereas pembrolizumab and nivolumab often exhibit irAEs beyond the first 3 months as treatment is discontinued. Nonetheless, the enduring toxicities of single-agent ipilimumab and PD-1 agents are similar in the long term [108].

## Novel Approaches to Immunotherapy

### New Checkpoint Targets

Druggable immune targets currently in the early stages of clinical investigation for novel ICIs include TIM-3, TIGIT, BTLA, VISTA and CD96 [112]. These targets may serve as the basis for novel ICI therapeutics in melanoma patients that become refractory to conventional ICIs. Alternatively, novel ICIs may offer better efficacy or toxicity profiles when combined with existing ICIs. 

TIM-3 is a type I transmembrane protein that belongs to the TIM family of immunoregulatory proteins that are found on many immune cells, including TILs. TIM-3 has been the most prominent of these as it has been suggested to regulate immune responses in autoimmunity and cancer [113]. TIM-3 is co-expressed with PD-1 on both CD4^+^ and CD8^+^ T-cells, and when both of their pathways are targeted together compared to either of them on their own, has been shown to be effective in controlling tumour growth, hence making TIM-3 an attractive immunotherapy candidate [114]. Moreover, TIM-3 is upregulated in patients that respond to anti-PD-1 therapy via the PI3K-Akt pathway [115]. Hence, TIM-3 and PD-1 co-blockade may restore T-cell responses more effectively than PD-1 monotherapy [116]. Currently anti-TIM-3 therapies are being investigated in combination with anti-PD-1 blockade in phase I/II trials (NCT04139902, NCT03708328) [47].

Another promising checkpoint candidate, B and T lymphocyte attenuator (BTLA) has been shown to play a role in the inhibition of CD8 + T cell expansion and function in melanoma [117]. In combination with PD-1 and TIM-3 blockade, inhibition of BTLA has been shown to reactivate dysfunctional melanoma antigen-specific CD8 + T cells, enhancing their proliferation and cytokine secretion. Icatolimab (JS004, Table 4), a new anti-BTLA mAb, is being tested in two phase-I trials for advanced solid tumours, alone or with PD-1 inhibitors (NCT04137900 and NCT04773951). In NCT04137900’s dose-expansion phase, 1/19 melanoma patients previously unresponsive to nivolumab and BRAF/MEK inhibitors achieved a partial response without severe adverse events [118].

Finally, VISTA (V-domain Ig suppressor of T cell activation) is a checkpoint ligand that is homologous to PD-L1 and suppresses T cell activation. In a study composed of 85 primary melanoma specimens, VISTA expression was shown to be correlated with decreases disease-specific survival (*p* = 0.05) and to be an independent negative prognostic factor (*p* = 0.02), suggesting its potential as an adjuvant immunotherapeutic intervention [119]. Thus, anti-VISTA mAbs have been studied in preclinical studies and recently in clinical-trial settings. In mice, CA-170, an oral PD-L1/L2 and VISTA dual inhibitor has been shown significant anti-tumour properties. In a phase I trial (NCT02812875), CA-170 showed acceptable safety and increased numbers of circulating activated CD4 + and CD8 + T cells [120]. Additionally, another phase I clinical trial (NCT05082610) is investigating the efficacy of monotherapy or combination therapies involving pembrolizumab with HMBD-002, an Fc-independent anti-VISTA monoclonal antibody, in patients with advanced solid tumours [118].

### Alternative Therapies

I) Cell therapies

Adoptive cell therapy (ACT) aims to deliver immune cells to destroy cancer cells. ACT involves the collection of a patient’s own immune cells, such as T cells, which are then engineered or activated in the laboratory to enhance their ability to target and destroy cancer cells. Once these modified or expanded cells are grown to sufficient numbers, they are infused back into the patient to mount an effective anti-tumour response [121].

Various forms of ACT are employed, each with distinct methodologies. These include: [1] TILs, these lymphocytes are cultivated from the tumour itself; [2] endogenous T Cell therapy, these are tumour-specific T cells cultured from the blood; [3] chimeric antigen receptor (CAR) T cells, these cells are engineered using a fusion of chimeric antibody and T cell receptor genes, which are then integrated into peripheral T cells; and [4] TCR transduced T Cells, this technique involves engineering T cell receptor genes to target the tumour, which are subsequently inserted into peripheral T cells [122].

The development of ACT was propelled by the description of IL-2 in 1976 which enabled ex vivo expansion of T lymphocytes while often preserving their effector function [123]. In 1985, Rosenberg et al., demonstrated that IL-2 administration could lead to complete durable tumour regressions in some patients with metastatic melanoma, prompting efforts to identify specific T cells and their target antigens responsible for this therapeutic effect [124]. By 1986, in vitro studies revealed that TILs extracted from resected melanomas included cells capable of specifically recognizing autologous tumours [125]. These findings culminated in a landmark 1988 study in which 20 patients with metastatic malignant melanoma were treated with TILs and IL-2 following a single intravenous dose of cyclophosphamide. Objective regression was observed in 60% of the 15 patients who had never been treated with IL-2 and in 40% of the 5 patients in which IL-2 therapies had previously failed. Regression of cancer was observed with duration of responses ranging from 2 to more than 13 months [126].

In the decades after the description of ACT using TILs, several small cohort phase 1/2 trials in advanced-stage melanoma were performed. These are summarised in a systematic review of 13 clinical trials including 410 patients with advanced-stage melanoma from 1988 to 2018 with results showing an ORR in the total cohort of 41%, ranging from 28% to 45%, with 14% complete responses and 27% partial responses [127].

Despite the potential of TIL therapy, clinical approval was delayed due to several factors, including the need for ongoing improvements in clinical response rates and the development and refinement of appropriate FDA good manufacturing guidelines for cell production [128].

Several key findings contributed to the development of ACT. Notably, it was discovered that administering IL-2 after cell infusion helps promote the survival and growth in vivo of the transferred cells in mouse tumour models, leading to the inclusion of IL-2 administration in the treatment regimen [128]. Additionally, in 2002, it was demonstrated that lymphodepletion using a nonmyeloablative chemotherapy regimen administered immediately before TIL transfer could enhance cancer regression and result in the persistent oligoclonal repopulation of the host with the transferred antitumour lymphocytes [129]. 

The FDA approved lifileucel (Amtagvi, Iovance Biotherapeutics, Inc.) in February 2024 based on data from a global, phase 2, multicohort, single-arm, C-144-01 trial (NCT02360579) in adult patients with advanced melanoma after progression on ICI and targeted therapies [130]. This trial included four cohorts. Approval was specifically based on data from a subset of 73 patients in the trial whose cancer had worsened despite treatment with a PD-1/PD-L1 ICI or a BRAK inhibitor and whose lifileucel dose was at least 7.5 billion cells. Results showed an ORR of 31.5% with a complete response in 3 (4.1%). Patients received a single intravenous infusion of lifileucel at a median dose of 21.1 × 10^9 viable cells following lymphodepletion with cyclophosphamide, mesna, and fludarabine. Post-infusion, IL-2 (aldesleukin) doses were administered to support in vivo cell expansion [128].

Lifileucel is approved for use in patients with advanced-stage melanoma refractory to a PD-1 blocking antibody, and in those with BRAFV600 mutations, BRAF/MEK inhibitors [131]. This immunotherapy employs the patient’s own TILs which are isolated from a surgically excised tumour. The TILs are subsequently expanded ex vivo using IL-2 and then reinfused into the patient to specifically target and eradicate tumour cells. The regimen involves a lymphodepleting regimen consisting of cyclophospimade (60 mg/kg daily with mesna for 2 days) followed by fludarabine (25 mg/m^2^ daily for 5 days). This is succeeded by a single lifileucel infusion (median dose 21.1 × 10^9 viable cells). Finally, by IL-2 (aldesleukin) is administered at 600,000 IU/kg every 8–12 h for up to 6 doses [132].

When data from cohorts 2 and 4 of the trial were combined (*n* = 153; pooled efficacy set), where patients had identical eligibility criteria, lifileucel manufacturing process, treatment regimen, and IRC response assessment, and received a single lifileucel infusion along with up to six doses of high-dose IL-2—the ORR was 31.4% [133].

Apart from Lifeleucel, other TIL products have demonstrated encouraging outcomes. In a multicentre phase 3 randomised trial of 168 patients with unresectable stage IIIC or IV melanoma, patients were assigned to receive either TIL or ipilimumab at 3 mg/kg at a 1:1 ratio. Administration of a minimum of 5 × 10^9 TILs was preceded by nonmyeloablative lymphodepleting chemotherapy (cyclophosphamide plus fludarabine) and succeeded by high-dose interleukin-2. Median PFS was 7.2 months (95% CI, 4.2 to 13.1) in patients treated with TILs compared to 3.1 months (95% CI, 3.0 to 4.3) with ipilimumab. OS was 54.3% (95% CI, 43.9 to 67.2) in the TIL group and 44.1% (95% CI, 33.6 to 57.8) in the ipilimumab group. Response rates were higher with TILs (49% vs. 21%). Grade 3–5 treatment related AEs were higher with TILs (100% vs. 57%) [134]. 

II) Combining Adoptive Cell Therapies and Immune Checkpoint Inhibitors

ACT has been investigated in combination with ICIs. In a trial by Mullinax et al. (2018) (NCT01701674) 12 patients with metastatic melanoma received four doses of ipilimumab (3 mg/kg) followed by chemotherapy, a TIL infusion and IL-2. Five patients (38.5%) experienced objective response, four of whom continued in objective response at 1 year and one of which became a complete response at 52 months. Median PFS was 7.3 months (95% CI 6.1–29.9 months) [135]. In another phase I clinical trial, patients with stage III/IV metastatic melanoma with unresectable disease were treated with TILs generated with anti-4-1BB agonistic antibody in vitro in combination with nivolumab infusions. 4/11 patients achieved a partial response (36% ORR) with a median PFS of 5 months [136]. Of note, a study exploring patient characteristics in 226 patients with metastatic melanoma treated with ACT found a decreased likelihood of response among patients who had previously undergone and were unresponsive to anti-PD-1 therapy (ORR of 56%, in patients naïve to anti-PD-1 therapy vs. 24% in patients refractory to anti-PD-1), supporting shared mechanisms of resistance in anti-PD-1 and ACT refractory disease [137]. 

III) Cancer Vaccines

Cancer vaccines are a promising therapeutic strategy, aiming to trigger anti-tumour specific immune response by utilising tumour antigens, leading to recognition and destruction of cancer cells [138]. Melanoma is particularly suitable for this type of treatment due to its high TMB, offering a wide array of antigens for vaccine development [139].

There are several types of cancer vaccines including: [1] vaccines based on heat shock proteins, gangliosides or peptides; [2] vaccines based on DNA/RNA; [3] whole cell vaccines; [4] vaccines based on dendritic cells; and [5] vaccines based on recombinant viruses [139].

Despite over a century of research, the success of therapeutic cancer vaccines has been inconsistent, especially in patients with advanced cancer. This inconsistency is attributed to several factors, including the heterogeneity of the tumour microenvironment, the presence of immunosuppressive cells, and the potential for tumours to develop escape mechanisms [140].

Nevertheless, several therapeutic vaccination strategies are under development and are being evaluated in preclinical and clinical trials. Notably, mRNA vaccines have been garnering significant attention, with UK trials in colorectal and pancreatic cancer launched as part of the Cancer Vaccine Launch Pad scheme [141].

KEYNOTE-942 is an open-label, randomised phase 2 trial in patients with completely resected, high-risk stage IIIB/C/D and IV cutaneous melanoma. 157 patients were randomised 2:1 to receive mRNA-4157 (1 mg every 3 weeks for a total of 9 doses) in combination with pembrolizumab (200 mg every 3 weeks for up to 18 cycles) or pembrolizumab alone. This study demonstrated that combination therapy significantly prolonged distant metastasis-free survival (DMFS) compared to monotherapy with pembrolizumab. The 18-month RFS rates were higher with combination therapy (78.6%, 95% CI: 69.0%-85.6%) compared to monotherapy (62.2%, 95% CI: 46.9%-74.3%). Combination therapy also showed a significant improvement in DMFS (HR = 0.347; 95% CI: 0.145–0.828; one-sided p-value 0.0063) and distant recurrence or death rates were lower with combination therapy (8.4%) compared to monotherapy (24%) [142]. Several biomarkers such as TMB, PD-L1 and circulating tumour DNA (ctDNA) might be predictive of outcomes to adjuvant treatment [143].

Several other ongoing trials are investigating ICI combinations with mRNA vaccines for melanoma (NCT04526899, NCT02410733, NCT03291002) with promising potential for clinical approval on the horizon. Combining cancer vaccines with ICIs leverages vaccines to boost immune cell activation and infiltration while ICIs sustain their function, potentially overcoming individual limitations for synergistic therapeutic effects [144].

IV) Oncolytic Viruses

Oncolytic viruses are engineered or naturally occurring to selectively infect and kill cancer cells. They work by directly targeting and destroying cancer cells often through a combination of viral replication within the tumour and stimulation of the immune system’s response against the tumour [145].

In 2015, the FDA approved T-VEC (tamoligene laherparepvec), a genetically altered herpes virus, for treating localized, inoperable metastatic stage IIIB/C-IVM1a melanoma. This represented a significant breakthrough, as T-VEC became the first oncolytic viral immunotherapy sanctioned for melanoma treatment [146].

The results that led to FDA approval originated from the OPTiM phase III trial of T-VEC in unresectable stage III-IV melanoma (*NCT00769704*), where 436 patients were randomised at a 2:1 ratio to receive either T-VEC or subcutaneous recombinant GM-CSF respectively. The T-VEC group exhibited notably higher durable response rate (DRR) and ORR compared to the GM-CSF group (DRR 16.3% vs. 2.1%, *p* < 0.001; ORR 26.4% vs. 5.7%, *p* < 0.001) [147]. Additionally, patients receiving T-VEC experienced extended median survival (23.3 vs. 18.9 months, *p* = 0.051). Notably, long-term follow-up analysis (median 49 months) confirms these initial results [122]. Further investigation suggests that early metastatic melanoma and lower tumour burden independently predict complete response attainment. The median time to achieve complete response in the T-VEC group was 8.6 months, correlating with improved OS [122].

Despite T-VEC’s inability to enhance OS when used alone, its combination with systemic therapy, particularly ICI, holds promise. Studies have shown improved overall response rates and systemic antitumour responses when T-VEC is combined with ICI. While recent trials like Masterkey-265, a phase 3 randomised double-blind multicentre study of T-VEC plus pembrolizumab, have not demonstrated significant PFS benefits, there remains interest in exploring T-VEC’s potential, especially for patients unable to tolerate or refractory to ICI, and those seeking palliative reduction in disease burden. The heterogeneity of advanced melanoma and responses to therapy underscore the ongoing importance of investigating T-VEC and other oncolytic viruses [148].

Additionally, the oncolytic-virus pipeline continues to expand. One notable example is RP1, a second-generation HSV-1-based oncolytic immunotherapy developed by Replimune Group, Inc. RP1 has been engineered by deleting the ICP34.5 and ICP47 genes and inserting the GALV-GP-R⁻ fusogenic protein along with human GM-CSF, with the aim of enhancing tumour-selective replication, tumour cell fusion/lysis and systemic immune activation [149]. Clinically, RP1 has been investigated in monotherapy and in combination with anti-PD-1 therapy (e.g., in the IGNYTE study) in patients with advanced melanoma and other solid tumours [150]. The FDA has granted Breakthrough Therapy Designation for RP1 in combination with nivolumab in anti-PD-1-refractory melanoma and a Biologics License Application was submitted under the Accelerated Approval pathway. These developments highlight RP1 as a promising next-generation oncolytic viral immunotherapy that, like T-VEC,- but with enhanced engineering, seeks to overcome limitations of first-generation agents in the checkpoint-resistant setting.

Furthermore, numerous additional oncolytic viruses are being explored in both preclinical studies and clinical trials. These include ONCOS-102 adenovirus, coxsackievirus A21, poliovirus and vesicular stomatitis virus (NCT03003676, NCT02307149, NCT04577807, NCT03865212) [123].

### Gut Microbiome Modulation

Finally, augmentation of the gut microbiome by inoculation of *Bifidobacteria* promotes CD8^+^ T-cell-mediated anti-cancer response in murine models of melanoma, while the combination of inoculation with *Bacteroides fragilis* and administration of anti-CTLA-4 ICIs decreased tumour growth in vivo [151]. Furthermore, faecal transplant of *Akkermansia muciniphila* has been associated with improved outcomes in anti-PD-1 ICI trials [152]. Faecal microbiota transplant in combination with immunotherapy in melanoma is undergoing investigation in phase I setting (NCT03772899). Similarly, antibiotics that alter the commensal gut flora decrease ICI efficacy across several cancer types [153, 154]. Thus, augmentation of the gut microbiome, either through inoculation or antibiotic treatment may offer a means of augmenting ICI efficacy. Ongoing trials, such as The Predicting Response to Immunotherapy for Melanoma with gut Microbiome and Metabolomics (PRIMM) trial (NCT03643289), a prospective cohort study will provide further insights into the intricate interplay between gut microbiota and immunotherapy response rates, paving the way for more personalized and effective treatment strategies.

## Conclusion

ICIs are promising anti-cancer immunotherapeutics with proven efficacy for the treatment of melanoma. Combination of nivolumab plus ipilimumab has become mainstay treatment for resistant melanoma with pembrolizumab or nivolumab monotherapy as second line where nivolumab plus ipilimumab is unsuitable or unacceptable due to factors such as heightened risk of severe adverse events, existing comorbid conditions, compromised overall health status, or patient preference for treatments with a more favourable side effect profile. However, combination nivolumab plus relatlimab is now being proposed as an alternative second line [50]. Although the combination of novel ICIs may overcome resistance to immune therapy by targeting pathways involved in ICI resistance [15, 155, 156], it may be necessary to design treatment protocols that incorporate sequential therapy approaches to mitigate the AEs associated with polytherapy [157].

Further research is required to improve the issues associated with this modality of anti-cancer therapy – particularly immunological resistance mechanisms, immune-related adverse events, and the sequence and dosing schedules for combinations of ICIs. The discovery and validation of predictive biomarkers in large prospective trials remains an important step in broadening the applications of ICI therapies by establishing patient subgroups which are most likely to experience a significant benefit. Combination therapy with chemotherapy, radiotherapy, targeted therapy, as well as gut microbiome modulation and other immune-based cancer treatments including novel adoptive immune cell therapies are promising fields of research that are coming of age and require further systematic investigation.

## Key References

Albrecht LJ, Livingstone E, Zimmer L, Schadendorf D. The Latest Option: Nivolumab and Relatlimab in Advanced Melanoma. Curr Oncol Rep. 2025 Jun;25 [6]:647–57.

- ○ Summarizes emerging data on dual checkpoint blockade with nivolumab (PD-1) and relatlimab (LAG-3) in advanced melanoma, highlighting efficacy, tolerability, and positioning relative to standard anti-PD-1 therapy.

Patel SP, Othus M, Chen Y, Wright GP, Yost KJ, Hyngstrom JR, et al. Neoadjuvant–Adjuvant or Adjuvant-Only Pembrolizumab in Advanced Melanoma. N Engl J Med. 2023 Mar 2;388 [9]:813–23.

- ○ This trial directly compares neoadjuvant + adjuvant vs. adjuvant-only use of pembrolizumab in high-risk patients, offering evidence that could reshape timing of checkpoint blockade in resected melanoma. It adds to the growing interest in neoadjuvant immunotherapy as a strategy to improve outcomes.

Seth R, Agarwala SS, Messersmith H, Alluri KC, Ascierto PA, Atkins MB, et al. Systemic Therapy for Melanoma: ASCO Guideline Update. J Clin Oncol. 2023 Oct 20;41 [30]:4794–820.

- ○ This guideline update synthesizes the latest evidence and offers updated recommendations on systemic therapy for melanoma, making it essential for clinicians and researchers to understand current standards of care and where gaps remain.

Wang SJ, Dougan SK, Dougan M. Immune mechanisms of toxicity from checkpoint inhibitors. Trends Cancer. 2023 Jul;9 [7]:543–53.

- ○ With broader use of checkpoint inhibitors, toxicity remains a key limitation. This paper clarifies emerging mechanistic understandings of immune‐related adverse events, which is critical for managing patients and developing safer therapies.

Wong SK, Blum SM, Sun X, Da Silva IP, Zubiri L, Ye F, et al. Efficacy and safety of immune checkpoint inhibitors in young adults with metastatic melanoma. Eur J Cancer. 2023 Mar 1;181:188–97.

- ○ Provides data specifically about younger adults, a subgroup often underrepresented in trials; helps define safety/efficacy in that age bracket and guides treatment expectations.

Li Y, Ju M, Miao Y, Zhao L, Xing L, Wei M. Advancement of anti-LAG ‐3 in cancer therapy. FASEB J. 2023 Nov;37 [11]:e23236.

- ○ LAG-3 is a newer checkpoint of interest; this review/paper integrates preclinical and clinical data around anti-LAG3 strategies, which are increasingly entering practice (e.g. relatlimab + nivolumab). Useful for understanding mechanisms and emerging therapeutic combinations.

Rosenberg SA. Lymphocytes as a living drug for cancer. Science. 2024 Jul 5;385(6704):25–6.

- ○ A perspective piece by a pioneer in adoptive cell therapy, reflecting on where “living drug” therapies (TILs, CAR-T etc.) stand, what the challenges are, especially for solid tumors, which frames the context for recent approvals.

Klobuch S, Seijkens TTP, Schumacher TN, Haanen JBAG. Tumour-infiltrating lymphocyte therapy for patients with advanced-stage melanoma. Nat Rev Clin Oncol [Internet]. 2024 Jan 8 [cited 2024 Aug 6]; Available from: "https://www.nature.com/articles/s41571-023-00848-w" https://www.nature.com/articles/s41571-023-00848-w

- ○ This is a recent, high-profile review of TIL (tumour-infiltrating lymphocyte) therapies at a time when such therapies (e.g. lifileucel) are moving into approval/use. It distills lessons, challenges, and likely paths forward for TIL in melanoma.

Khattak A, Weber JS, Meniawy T, Taylor MH, Ansstas G, Kim KB, et al. Distant metastasis-free survival results from the randomized, phase 2 mRNA-4157-P201/KEYNOTE-942 trial. J Clin Oncol. 2023 Jun 10;41(17_suppl): LBA9503–LBA9503.

- ○ Reports updated distant metastasis-free survival (DMFS) results from the KEYNOTE-942 trial, confirming that the personalized neoantigen vaccine mRNA-4157 (V940) plus pembrolizumab significantly reduces risk of recurrence compared to pembrolizumab alone.

Weber JS, Carlino MS, Khattak A, Meniawy T, Ansstas G, Taylor MH, et al. Individualised neoantigen therapy mRNA-4157 (V940) plus pembrolizumab versus pembrolizumab monotherapy in resected melanoma (KEYNOTE-942): a randomised, phase 2b study. Lancet Lond Engl. 2024 Feb 17;403(10427):632–44.

- ○ This study is among the first randomized controlled trials showing benefit of a personalized neoantigen vaccine + PD-1 blockade in the adjuvant setting, which is a significant conceptual advance in melanoma immunotherapy.

## Data Availability

No datasets were generated or analysed during the current study.

## References

[1] Ali Z, Yousaf N, Larkin J. Melanoma epidemiology, biology and prognosis. EJC Suppl. 2013;11(2):81–91. 26217116 10.1016/j.ejcsup.2013.07.012 PMC4041476

[2] Liu Y, Sheikh MS. Melanoma: molecular pathogenesis and therapeutic management. Mol Cell Pharmacol. 2014;6(3):228. 25745537 PMC4346328

[3] Miller AJ, Mihm MC, Melanoma. N Engl J Med. 2006;355(1):51–65. 16822996 10.1056/NEJMra052166

[4] SEER*Explorer: An interactive website for SEER cancer statistics [Internet]. Surveillance Research Program, National Cancer Institute. 2024 Apr 17. [cited 2024 May 10]. Available from: https://seer.cancer.gov/statistics-network/explorer/. Data source(s): SEER Incidence Data, November 2023 Submission (1975–2021), SEER 22 registries (excluding Illinois and Massachusetts). Expected Survival Life Tables by Socio-Economic Standards.

[5] Kosary CL, Altekruse SF, Ruhl J, Lee R, Dickie L. Clinical and prognostic factors for melanoma of the skin using SEER registries: collaborative stage data collection system, version 1 and version 2. Cancer. 2014;120(Suppl 23):3807–14. 25412392 10.1002/cncr.29050

[6] Geller J, Swetter SM, Leyson J, Miller DR, Brooks K, Geller AC. Crafting a melanoma educational campaign to reach Middle-Aged and older men. J Cutan Med Surg. 2006;10(6):259–68. 17241595 10.2310/7750.2006.00066

[7] Beddingfield FC. The melanoma epidemic: *Res Ipsa loquitur*. Oncologist. 2003;8(5):459–65. 14530499 10.1634/theoncologist.8-5-459

[8] Conforti C, Zalaudek I. Epidemiology and risk factors of melanoma: A review. Dermatol Pract Concept. 2021;11(Suppl 1):e2021161S. 34447610 10.5826/dpc.11S1a161S PMC8366310

[9] Erdei E, Torres SM. A new Understanding in the epidemiology of melanoma. Expert Rev Anticancer Ther. 2010;10(11):1811. 21080806 10.1586/era.10.170 PMC3074354

[10] Califano J, Nance M. Malignant melanoma. Facial Plast Surg Clin N Am. 2009;17(3):337–48. 10.1016/j.fsc.2009.05.002 19698915

[11] Chalmers ZR, Connelly CF, Fabrizio D, Gay L, Ali SM, Ennis R, et al. Analysis of 100,000 human cancer genomes reveals the landscape of tumor mutational burden. Genome Med. 2017;9(1):34. 28420421 10.1186/s13073-017-0424-2 PMC5395719

[12] Transcriptional downregulation of. MHC class I and melanoma de- differentiation in resistance to PD-1 inhibition | Nature Communications [Internet]. [cited 2021 May 16]. Available from: https://www.nature.com/articles/s41467-020-15726-7 10.1038/s41467-020-15726-7 PMC7171183 32312968

[13] Petrova V, Arkhypov I, Weber R, Groth C, Altevogt P, Utikal J, et al. Modern aspects of immunotherapy with checkpoint inhibitors in melanoma. Int J Mol Sci. 2020;21(7):2367. 32235439 10.3390/ijms21072367 PMC7178114

[14] Seidel JA, Otsuka A, Kabashima K. Anti-PD-1 and Anti-CTLA-4 Therapies in Cancer: Mechanisms of Action, Efficacy, and Limitations. Front Oncol [Internet]. 2018 Mar 28 [cited 2019 Oct 14];8. Available from: https://www.ncbi.nlm.nih.gov/pmc/articles/PMC5883082/10.3389/fonc.2018.00086 PMC5883082 29644214

[15] Nicholson LB. The immune system. Essays Biochem. 2016;60(3):275–301. 27784777 10.1042/EBC20160017 PMC5091071

[16] Chaplin DD. Overview of the immune response. J Allergy Clin Immunol. 2010;125(2 Suppl 2):S3–23. 20176265 10.1016/j.jaci.2009.12.980 PMC2923430

[17] Chi H, Pepper M, Thomas PG. Principles and therapeutic applications of adaptive immunity. Cell. 2024;187(9):2052–78. 38670065 10.1016/j.cell.2024.03.037 PMC11177542

[18] Xing Y, Hogquist KA. T-Cell Tolerance: Central and Peripheral. Cold Spring Harb Perspect Biol [Internet]. 2012 Jun [cited 2019 Dec 9];4(6). Available from: https://www.ncbi.nlm.nih.gov/pmc/articles/PMC3367546/10.1101/cshperspect.a006957 PMC3367546 22661634

[19] Frontiers. | Twenty Years of AIRE | Immunology [Internet]. [cited 2021 May 16]. Available from: https://www.frontiersin.org/articles/10.3389/fimmu.2018.00098/full.

[20] Regulation of T cell. expansion by antigen presentation dynamics | PNAS [Internet]. [cited 2021 May 16]. Available from: https://www.pnas.org/content/116/13/5914

[21] ElTanbouly MA, Noelle RJ. Rethinking peripheral T cell tolerance: checkpoints across a T cell’s journey. Nat Rev Immunol. 2021;21(4):257–67. 33077935 10.1038/s41577-020-00454-2 PMC12536352

[22] Yun TJ, Clark EA, Cooperation. Mechanisms of Cellular. In: Delves PJ, editor. Encyclopedia of Immunology (Second Edition) [Internet]. Oxford: Elsevier; 1998 [cited 2019 Dec 9]. pp. 651–6. Available from: http://www.sciencedirect.com/science/article/pii/B0122267656001791

[23] Ford ML, Adams AB, Pearson TC. Targeting co-stimulatory pathways: transplantation and autoimmunity. Nat Rev Nephrol. 2014;10(1):14–24. 24100403 10.1038/nrneph.2013.183 PMC4365450

[24] Rallis KS, Hillyar CRT, Sideris M, Davies JK. T-cell-based immunotherapies for haematological Cancers, part A: A SWOT analysis of immune checkpoint inhibitors (ICIs) and bispecific T-Cell engagers (BiTEs). Anticancer Res. 2021;41(3):1123–41. 33788704 10.21873/anticanres.14870

[25] Mariuzza RA, Shahid S, Karade SS. The immune checkpoint receptor LAG3: Structure, function, and target for cancer immunotherapy. J Biol Chem. 2024;300(5):107241. 38556085 10.1016/j.jbc.2024.107241 PMC11061240

[26] Ledbetter JA, Linsley PS. CD28. In: Delves PJ, editor. Encyclopedia of Immunology (Second Edition) [Internet]. Oxford: Elsevier; 1998 [cited 2019 Dec 9]. pp. 482–3. Available from: http://www.sciencedirect.com/science/article/pii/B0122267656001316

[27] Mir MA. Chapter 1 - Introduction to Costimulation and Costimulatory Molecules. In: Mir MA, editor. Developing Costimulatory Molecules for Immunotherapy of Diseases [Internet]. Academic Press; 2015 [cited 2019 Dec 9]. pp. 1–43. Available from: http://www.sciencedirect.com/science/article/pii/B9780128025857000017

[28] van Nieuwenhuijze A, Liston A. Chapter Four - The Molecular Control of Regulatory T Cell Induction. In: Liston A, editor. Progress in Molecular Biology and Translational Science [Internet]. Academic Press; 2015 [cited 2019 Dec 9]. pp. 69–97. (Regulatory T Cells in Health and Disease; vol. 136). Available from: http://www.sciencedirect.com/science/article/pii/S1877117315001908 10.1016/bs.pmbts.2015.09.001 26615093

[29] Singh SS, Jois SD. Chapter One - Homo- and Heterodimerization of Proteins in Cell Signaling: Inhibition and Drug Design. In: Donev R, editor. Advances in Protein Chemistry and Structural Biology [Internet]. Academic Press; 2018 [cited 2019 Dec 9]. pp. 1–59. (Protein-Protein Interactions in Human Disease, Part B; vol. 111). Available from: http://www.sciencedirect.com/science/article/pii/S1876162317300640 10.1016/bs.apcsb.2017.08.003 PMC6203451 29459028

[30] Sanchez-Lockhart M, Rojas AV, Fettis MM, Bauserman R, Higa TR, Miao H, et al. T cell receptor signaling can directly enhance the avidity of CD28 ligand binding. PLoS ONE. 2014;9(2):e89263. 24586641 10.1371/journal.pone.0089263 PMC3933428

[31] Granier C, Guillebon ED, Blanc C, Roussel H, Badoual C, Colin E et al. Mechanisms of action and rationale for the use of checkpoint inhibitors in cancer. ESMO Open [Internet]. 2017 Jul 1 [cited 2019 Dec 9];2(2). Available from: https://esmoopen.bmj.com/content/2/2/e000213 10.1136/esmoopen-2017-000213 PMC5518304 28761757

[32] Bell RB, Feng Z, Bifulco CB, Leidner R, Weinberg A, Fox BA. 15 - Immunotherapy. In: Bell RB, Fernandes RP, Andersen PE, editors. Oral, Head and Neck Oncology and Reconstructive Surgery [Internet]. Elsevier; 2018 [cited 2019 Dec 9]. pp. 314–40. Available from: http://www.sciencedirect.com/science/article/pii/B9780323265683000154

[33] Freeman GJ. Structures of PD-1 with its ligands: sideways and dancing cheek to cheek. Proc Natl Acad Sci U S A. 2008;105(30):10275–6. 18650389 10.1073/pnas.0805459105 PMC2492504

[34] Pardoll D. 52 - Cancer Immunotherapy with Vaccines and Checkpoint Blockade. In: Mendelsohn J, Gray JW, Howley PM, Israel MA, Thompson CB, editors. The Molecular Basis of Cancer (Fourth Edition) [Internet]. Philadelphia: Content Repository Only! 2015 [cited 2019 Dec 9]. pp. 709–738.e8. Available from: http://www.sciencedirect.com/science/article/pii/B9781455740666000524

[35] Chen L, Flies DB. Molecular mechanisms of T cell co-stimulation and co-inhibition. Nat Rev Immunol. 2013;13(4):227–42. 23470321 10.1038/nri3405 PMC3786574

[36] Callahan MK, Postow MA, Wolchok JD. Chapter 19 - Antibodies to Stimulate Host Immunity: Lessons from Ipilimumab. In: Prendergast GC, Jaffee EM, editors. Cancer Immunotherapy (Second Edition) [Internet]. San Diego: Academic Press; 2013 [cited 2019 Dec 9]. pp. 287–307. Available from: http://www.sciencedirect.com/science/article/pii/B9780123942968000191

[37] Ikemizu S, Davis SJ. Chapter 2 - Principles of Protein Recognition by Small T-Cell Adhesion Proteins and Costimulatory Receptors. In: Putterman C, Cowburn D, Almo S, editors. Structural Biology in Immunology [Internet]. Academic Press; 2018 [cited 2019 Dec 9]. pp. 39–80. Available from: http://www.sciencedirect.com/science/article/pii/B9780128033692000024

[38] Sekhon N, Kumbla RA, Mita M. Chapter 1 - Current Trends in Cancer Therapy. In: Gottlieb RA, Mehta PK, editors. Cardio-Oncology [Internet]. Boston: Academic Press; 2017 [cited 2019 Dec 9]. pp. 1–24. Available from: http://www.sciencedirect.com/science/article/pii/B978012803547400001X

[39] Mir MA. Chapter 5 - Costimulation in Lymphomas and Cancers. In: Mir MA, editor. Developing Costimulatory Molecules for Immunotherapy of Diseases [Internet]. Academic Press; 2015 [cited 2019 Dec 9]. pp. 185–254. Available from: http://www.sciencedirect.com/science/article/pii/B9780128025857000054

[40] Joshi H, Press MF. 22 - Molecular Oncology of Breast Cancer. In: Bland KI, Copeland EM, Klimberg VS, Gradishar WJ, editors. The Breast (Fifth Edition) [Internet]. Elsevier; 2018 [cited 2019 Dec 9]. pp. 282–307.e5. Available from: http://www.sciencedirect.com/science/article/pii/B9780323359559000222

[41] Buchbinder EI, Desai A. CTLA-4 and PD-1 pathways. Am J Clin Oncol. 2016;39(1):98–106. 26558876 10.1097/COC.0000000000000239 PMC4892769

[42] Khalil DN, Budhu S, Gasmi B, Zappasodi R, Hirschhorn-Cymerman D, Plitt T et al. Chapter One - The New Era of Cancer Immunotherapy: Manipulating T-Cell Activity to Overcome Malignancy. In: Wang XY, Fisher PB, editors. Advances in Cancer Research [Internet]. Academic Press; 2015 [cited 2019 Dec 9]. pp. 1–68. (Immunotherapy of Cancer; vol. 128). Available from: http://www.sciencedirect.com/science/article/pii/S0065230X1500038X 10.1016/bs.acr.2015.04.010 26216629

[43] Marasco M, Berteotti A, Weyershaeuser J, Thorausch N, Sikorska J, Krausze J, et al. Molecular mechanism of SHP2 activation by PD-1 stimulation. Sci Adv. 2020;6(5):eaay4458. 32064351 10.1126/sciadv.aay4458 PMC6994217

[44] Chocarro L, Blanco E, Zuazo M, Arasanz H, Bocanegra A, Fernández-Rubio L, et al. Understanding LAG-3 signaling. Int J Mol Sci. 2021;22(10):5282. 34067904 10.3390/ijms22105282 PMC8156499

[45] Andrews LP, Cillo AR, Karapetyan L, Kirkwood JM, Workman CJ, Vignali DAA. Molecular pathways and mechanisms of LAG3 in cancer therapy. Clin Cancer Res Off J Am Assoc Cancer Res. 2022;28(23):5030–9. 10.1158/1078-0432.CCR-21-2390 PMC9669281 35579997

[46] Rodríguez-Guilarte L, Ramírez MA, Andrade CA, Kalergis AM. LAG-3 contribution to T cell downmodulation during acute respiratory viral infections. Viruses. 2023;15(1):147. 36680187 10.3390/v15010147 PMC9865459

[47] Zila N, Hoeller C, Paulitschke V. Novel immune checkpoints beyond PD-1 in advanced melanoma. Memo - Mag Eur Med Oncol. 2021;14(2):135–42.

[48] Vinay DS, Ryan EP, Pawelec G, Talib WH, Stagg J, Elkord E, et al. Immune evasion in cancer: mechanistic basis and therapeutic strategies. Semin Cancer Biol. 2015;35:S185–98. 25818339 10.1016/j.semcancer.2015.03.004

[49] Hanahan D, Weinberg RA. Hallmarks of cancer: the next generation. Cell. 2011;144(5):646–74. 21376230 10.1016/j.cell.2011.02.013

[50] Li R, Qiu J, Zhang Z, Qu C, Tang Z, Yu W, et al. Prognostic significance of Lymphocyte-activation gene 3 (LAG3) in patients with solid tumors: a systematic review, meta-analysis and pan-cancer analysis. Cancer Cell Int. 2023;23(1):306. 38041068 10.1186/s12935-023-03157-5 PMC10693146

[51] Hadash-Bengad R, Hajaj E, Klein S, Merims S, Frank S, Eisenberg G et al. Immunotherapy Potentiates the Effect of Chemotherapy in Metastatic Melanoma—A Retrospective Study. Front Oncol [Internet]. 2020 Feb 14 [cited 2021 Mar 9];10. Available from: https://www.ncbi.nlm.nih.gov/pmc/articles/PMC7033746/10.3389/fonc.2020.00070 PMC7033746 32117727

[52] Nivolumab–relatlimab for untreated unresectable or metastatic melanoma in people 12 years and over (2024) NICE technology appraisal 950. 40198032

[53] Vaddepally RK, Kharel P, Pandey R, Garje R, Chandra AB. Review of indications of FDA-Approved immune checkpoint inhibitors per NCCN guidelines with the level of evidence. Cancers. 2020;12(3):738. 32245016 10.3390/cancers12030738 PMC7140028

[54] Shah V, Panchal V, Shah A, Vyas B, Agrawal S, Bharadwaj S. Immune checkpoint inhibitors in metastatic melanoma therapy (Review). Med Int. 2024;4(2):13. 10.3892/mi.2024.137 PMC10895472 38410760

[55] Weiss SA, Wolchok JD, Sznol M. Immunotherapy of melanoma: facts and hopes. Clin Cancer Res. 2019;25(17):5191–201. 30923036 10.1158/1078-0432.CCR-18-1550 PMC6726509

[56] Huang AC, Zappasodi R. A decade of checkpoint Blockade immunotherapy in melanoma: Understanding the molecular basis for immune sensitivity and resistance. Nat Immunol. 2022;23(5):660–70. 35241833 10.1038/s41590-022-01141-1 PMC9106900

[57] Atkins MB, Kunkel L, Sznol M, Rosenberg SA. High-dose Recombinant interleukin-2 therapy in patients with metastatic melanoma: long-term survival update. Cancer J Sci Am. 2000;6(Suppl 1):S11–14. 10685652

[58] McDermott D, Haanen J, Chen TT, Lorigan P, O’Day S. Efficacy and safety of ipilimumab in metastatic melanoma patients surviving more than 2 years following treatment in a phase III trial (MDX010-20). Ann Oncol. 2013;24(10):2694–8. 23942774 10.1093/annonc/mdt291

[59] Eggermont AMM, Chiarion-Sileni V, Grob JJ, Dummer R, Wolchok JD, Schmidt H, et al. Prolonged survival in stage III melanoma with ipilimumab adjuvant therapy. N Engl J Med. 2016;375(19):1845–55. 27717298 10.1056/NEJMoa1611299 PMC5648545

[60] National Library of Medicine. Study of pembrolizumab (MK-3475) versus ipilimumab in participants with advanced melanoma (MK-3475-006/KEYNOTE-006) [Internet]. ClinicalTrials.gov; [cited 2025 Feb 6]. Available from: https://clinicaltrials.gov/study/NCT01295827

[61] Hamid O, Robert C, Daud A, Hodi FS, Hwu WJ, Kefford R, et al. Five-year survival outcomes for patients with advanced melanoma treated with pembrolizumab in KEYNOTE-001. Ann Oncol Off J Eur Soc Med Oncol. 2019;01(4):582–8. 10.1093/annonc/mdz011 PMC6503622 30715153

[62] Robert C, Schachter J, Long GV, Arance A, Grob JJ, Mortier L et al. 10.1056/NEJMoa1503093. 2015 [cited 2019 Dec 9]. Pembrolizumab versus Ipilimumab in Advanced Melanoma. Available from: https://www.nejm.org/doi/10.1056/NEJMoa1503093 10.1056/NEJMoa1503093 25891173

[63] Eggermont AMM, Blank CU, Mandala M, Long GV, Atkinson V, Dalle S, et al. Adjuvant pembrolizumab versus placebo in resected stage III melanoma. N Engl J Med. 2018;378(19):1789–801. 29658430 10.1056/NEJMoa1802357

[64] Ottaviano M, De Placido S, Ascierto PA. Recent success and limitations of immune checkpoint inhibitors for cancer: a lesson from melanoma. Virchows Arch. 2019;474(4):421–32. 30747264 10.1007/s00428-019-02538-4

[65] Weber JS, D’Angelo SP, Minor D, Hodi FS, Gutzmer R, Neyns B, et al. Nivolumab versus chemotherapy in patients with advanced melanoma who progressed after anti-CTLA-4 treatment (CheckMate 037): a randomised, controlled, open-label, phase 3 trial. Lancet Oncol. 2015;16(4):375–84. 25795410 10.1016/S1470-2045(15)70076-8

[66] Hodi FS, O’Day SJ, McDermott DF, Weber RW, Sosman JA, Haanen JB, et al. Improved survival with ipilimumab in patients with metastatic melanoma. N Engl J Med. 2010;363(8):711–23. 20525992 10.1056/NEJMoa1003466 PMC3549297

[67] Callahan MK, Kluger H, Postow MA, Segal NH, Lesokhin A, Atkins MB, et al. Nivolumab plus ipilimumab in patients with advanced melanoma: updated Survival, Response, and safety data in a phase I Dose-Escalation study. J Clin Oncol. 2017;36(4):391–8. 29040030 10.1200/JCO.2017.72.2850 PMC5946731

[68] Hodi FS, Chesney J, Pavlick AC, Robert C, Grossmann KF, McDermott DF, et al. Combined nivolumab and ipilimumab versus ipilimumab alone in patients with advanced melanoma: 2-year overall survival outcomes in a multicentre, randomised, controlled, phase 2 trial. Lancet Oncol. 2016;17(11):1558–68. 27622997 10.1016/S1470-2045(16)30366-7 PMC5630525

[69] Wolchok JD, Chiarion-Sileni V, Gonzalez R, Rutkowski P, Grob JJ, Cowey CL, et al. Overall survival with combined nivolumab and ipilimumab in advanced melanoma. N Engl J Med. 2017;377(14):1345–56. 28889792 10.1056/NEJMoa1709684 PMC5706778

[70] Albrecht LJ, Livingstone E, Zimmer L, Schadendorf D. The latest option: nivolumab and relatlimab in advanced melanoma. Curr Oncol Rep. 2023;25(6):647–57. 37004702 10.1007/s11912-023-01406-4 PMC10164023

[71] Pradeep J, Win TT, Aye SN, Sreeramareddy CT. Efficacy and safety of immune checkpoint inhibitors for advanced malignant melanoma: A Meta-Analysis on monotherapy vs combination therapy. J Cancer. 2022;13(10):3091–102. 36046644 10.7150/jca.72210 PMC9414012

[72] Responses to Pembrolizumab and Ipilimumab After Anti–PD-1/L1 Failure in Advanced Melanoma. - The ASCO Post [Internet]. [cited 2021 Jul 9]. Available from: https://ascopost.com/news/july-2021/responses-to-pembrolizumab-and-ipilimumab-after-anti-pd-1l1-failure-in-advanced-melanoma/?utm_source=TAP%2DEN%2D070221%2DINTL&utm_medium=email&utm_term=a418f600fc692d3db14763f364e82bbc?bc_md5=a418f600fc692d3db14763f364e82bbc

[73] Olson DJ, Eroglu Z, Brockstein B, Poklepovic AS, Bajaj M, Babu S et al. Pembrolizumab plus ipilimumab following Anti-PD-1/L1 failure in melanoma. J Clin Oncol Off J Am Soc Clin Oncol. 2021;JCO2100079. 10.1200/JCO.21.00079 PMC8376314 33945288

[74] Pires da Silva I, Ahmed T, Reijers ILM, Weppler AM, Betof Warner A, Patrinely JR, et al. Ipilimumab alone or ipilimumab plus anti-PD-1 therapy in patients with metastatic melanoma resistant to anti-PD-(L)1 monotherapy: a multicentre, retrospective, cohort study. Lancet Oncol. 2021;22(6):836–47. 33989557 10.1016/S1470-2045(21)00097-8

[75] VanderWalde A, Bellasea SL, Kendra KL, Khushalani NI, Campbell KM, Scumpia PO, et al. Ipilimumab with or without nivolumab in PD1/PDL1 Blockade refractory metastatic melanoma: a randomized phase 2 trial. Nat Med. 2023;29(9):2278–85. 37592104 10.1038/s41591-023-02498-y PMC10708907

[76] Ascierto PA, Melero I, Bhatia S, Bono P, Sanborn RE, Lipson EJ, et al. Initial efficacy of anti-lymphocyte activation gene-3 (anti–LAG-3; BMS-986016) in combination with nivolumab (nivo) in Pts with melanoma (MEL) previously treated with anti–PD-1/PD-L1 therapy. J Clin Oncol. 2017;35(15suppl):9520–9520.

[77] Frampton AE, Sivakumar S. A new combination immunotherapy in advanced melanoma. N Engl J Med. 2022;386(1):91–2. 34986291 10.1056/NEJMe2116892

[78] Chavanton A, Mialhe F, Abrey J, Baeza Garcia A, Garrido C. LAG-3 : recent developments in combinational therapies in cancer. Cancer Sci. 2024 Aug;115(8):2494-2505. 10.1111/cas.16205 PMC11309939 38702996

[79] Hieken TJ, Kreidieh F, Aedo-Lopez V, Block MS, McArthur GA, Amaria RN. Neoadjuvant immunotherapy in melanoma: the paradigm shift. Am Soc Clin Oncol Educ Book. 2023; 43:e390614. 10.1200/EDBK_390614 37116111

[80] Patel SP, Othus M, Chen Y, Wright GP, Yost KJ, Hyngstrom JR, et al. Neoadjuvant–Adjuvant or Adjuvant-Only pembrolizumab in advanced melanoma. N Engl J Med. 2023;388(9):813–23. 36856617 10.1056/NEJMoa2211437 PMC10410527

[81] Seth R, Agarwala SS, Messersmith H, Alluri KC, Ascierto PA, Atkins MB, et al. Systemic therapy for melanoma: ASCO guideline update. J Clin Oncol. 2023;41(30):4794–820. 37579248 10.1200/JCO.23.01136

[82] Blank CU, Rozeman EA, Fanchi LF, Sikorska K, van de Wiel B, Kvistborg P, et al. Neoadjuvant versus adjuvant ipilimumab plus nivolumab in macroscopic stage III melanoma. Nat Med. 2018;24(11):1655–61. 30297911 10.1038/s41591-018-0198-0

[83] Blank CU, Lucas MW, Scolyer RA, van de Wiel BA, Menzies AM, Lopez-Yurda M, et al. Neoadjuvant nivolumab and ipilimumab in resectable stage III melanoma. N Engl J Med. 2024;391(18):1696–708. 38828984 10.1056/NEJMoa2402604

[84] Forde PM, Spicer J, Lu S, Provencio M, Mitsudomi T, Awad MM, et al. Neoadjuvant nivolumab plus chemotherapy in resectable lung cancer. N Engl J Med. 2022;386(21):1973–85. 35403841 10.1056/NEJMoa2202170 PMC9844511

[85] Rallis KS, Yau THL, Sideris M. Chemoradiotherapy in cancer treatment: rationale and clinical applications. Anticancer Res. 2021;41(1):1–7. 33419794 10.21873/anticanres.14746

[86] Reits EA, Hodge JW, Herberts CA, Groothuis TA, Chakraborty M, Wansley EK, et al. Radiation modulates the peptide repertoire, enhances MHC class I expression, and induces successful antitumor immunotherapy. J Exp Med. 2006;203(5):1259–71. 16636135 10.1084/jem.20052494 PMC3212727

[87] Golden EB, Frances D, Pellicciotta I, Demaria S, Helen Barcellos-Hoff M, Formenti SC. Radiation fosters dose-dependent and chemotherapy-induced Immunogenic cell death. Oncoimmunology. 2014;3:e28518. 25071979 10.4161/onci.28518 PMC4106151

[88] Postow MA, Callahan MK, Barker CA, Yamada Y, Yuan J, Kitano S, et al. Immunologic correlates of the abscopal effect in a patient with melanoma. N Engl J Med. 2012;366(10):925–31. 22397654 10.1056/NEJMoa1112824 PMC3345206

[89] Rallis KS, Hillyar CRT, Sideris M, Davies JK. T-cell-based immunotherapies for haematological Cancers, part B: A SWOT analysis of adoptive cell therapies. Anticancer Res. 2021;41(3):1143–56. 33788705 10.21873/anticanres.14871

[90] Robert C, Thomas L, Bondarenko I, O’Day S, Weber J, Garbe C, et al. Ipilimumab plus Dacarbazine for previously untreated metastatic melanoma. N Engl J Med. 2011;364(26):2517–26. 21639810 10.1056/NEJMoa1104621

[91] Tang C, Wang X, Soh H, Seyedin S, Cortez MA, Krishnan S, et al. Combining radiation and immunotherapy: a new systemic therapy for solid tumors? Cancer Immunol Res. 2014;2(9):831–8. 25187273 10.1158/2326-6066.CIR-14-0069 PMC5367158

[92] Weber J, Hamid O, Amin A, O’Day S, Masson E, Goldberg SM, et al. Randomized phase I Pharmacokinetic study of ipilimumab with or without one of two different chemotherapy regimens in patients with untreated advanced melanoma. Cancer Immun. 2013;13:7. 23833564 PMC3700777

[93] Vera Aguilera J, Paludo J, McWilliams RR, Zhang H, Li Y, Kumar AB, et al. Chemo-immunotherapy combination after PD-1 inhibitor failure improves clinical outcomes in metastatic melanoma patients. Melanoma Res. 2020;30(4):364–75. 32404734 10.1097/CMR.0000000000000669 PMC7331824

[94] Goodman RS, Jung S, Quintos J, Johnson DB. Therapeutic responses to combination nivolumab and Temozolomide as salvage therapy for metastatic melanoma: A case series. Oncologist. 2023;28(9):e839–42. 37338166 10.1093/oncolo/oyad184 PMC10485291

[95] Koller KM, Mackley HB, Liu J, Wagner H, Talamo G, Schell TD, et al. Improved survival and complete response rates in patients with advanced melanoma treated with concurrent ipilimumab and radiotherapy versus ipilimumab alone. Cancer Biol Ther. 2017;18(1):36–42. 27905824 10.1080/15384047.2016.1264543 PMC5323007

[96] Knispel S, Stang A, Zimmer L, Lax H, Gutzmer R, Heinzerling L, et al. Impact of a preceding radiotherapy on the outcome of immune checkpoint Inhibition in metastatic melanoma: a multicenter retrospective cohort study of the DeCOG. J Immunother Cancer. 2020;8(1):e000395. 32371460 10.1136/jitc-2019-000395 PMC7228559

[97] Ziogas DC, Theocharopoulos C, Koutouratsas T, Haanen J, Gogas H. Mechanisms of resistance to immune checkpoint inhibitors in melanoma: what we have to overcome? Cancer Treat Rev. 2023;113:102499. 36542945 10.1016/j.ctrv.2022.102499

[98] Vukadin S, Khaznadar F, Kizivat T, Vcev A, Smolic M. Molecular mechanisms of resistance to immune checkpoint inhibitors in melanoma treatment: an update. Biomedicines. 2021;9(7):835. 34356899 10.3390/biomedicines9070835 PMC8301472

[99] Knight A, Karapetyan L, Kirkwood JM. Immunotherapy in melanoma: recent advances and future directions. Cancers. 2023;15(4):1106. 36831449 10.3390/cancers15041106 PMC9954703

[100] Carlino MS, Larkin J, Long GV. Immune checkpoint inhibitors in melanoma. Lancet. 2021;398(10304):1002–14. 34509219 10.1016/S0140-6736(21)01206-X

[101] Chennamadhavuni A, Abushahin L, Jin N, Presley CJ, Manne A. Risk factors and biomarkers for immune-Related adverse events: A practical guide to identifying High-Risk patients and rechallenging immune checkpoint inhibitors. Front Immunol. 2022;13:779691. 35558065 10.3389/fimmu.2022.779691 PMC9086893

[102] Wang SJ, Dougan SK, Dougan M. Immune mechanisms of toxicity from checkpoint inhibitors. Trends Cancer. 2023;9(7):543–53. 37117135 10.1016/j.trecan.2023.04.002 PMC10330206

[103] Johnson DB, Nebhan CA, Moslehi JJ, Balko JM. Immune-checkpoint inhibitors: long-term implications of toxicity. Nat Rev Clin Oncol. 2022;19(4):254–67. 35082367 10.1038/s41571-022-00600-w PMC8790946

[104] Goodman RS, Jung S, Balko JM, Johnson DB. Biomarkers of immune checkpoint inhibitor response and toxicity: challenges and opportunities. Immunol Rev. 2023;318(1):157–66. 37470280 10.1111/imr.13249 PMC10528475

[105] Bajetta E, Del Vecchio M, Bernard-Marty C, Vitali M, Buzzoni R, Rixe O, et al. Metastatic melanoma: chemotherapy. Semin Oncol. 2002;29(5):427–45. 12407508 10.1053/sonc.2002.35238

[106] Schwartzentruber DJ. Guidelines for the safe administration of high-dose interleukin-2. J Immunother Hagerstown Md 1997. 2001;24(4):287–93. 10.1097/00002371-200107000-00004 11565830

[107] Wong SK, Blum SM, Sun X, Da Silva IP, Zubiri L, Ye F, et al. Efficacy and safety of immune checkpoint inhibitors in young adults with metastatic melanoma. Eur J Cancer. 2023;181:188–97. 36680880 10.1016/j.ejca.2022.12.013

[108] Aroldi F, Middleton MR. Long-Term outcomes of immune checkpoint Inhibition in metastatic melanoma. Am J Clin Dermatol. 2022;23(3):331–8. 35359259 10.1007/s40257-022-00681-4

[109] Wang DY, Salem JE, Cohen JV, Chandra S, Menzer C, Ye F, et al. Fatal toxic effects associated with immune checkpoint inhibitors: A systematic review and Meta-analysis. JAMA Oncol. 2018;4(12):1721–8. 30242316 10.1001/jamaoncol.2018.3923 PMC6440712

[110] Tawbi HA, Schadendorf D, Lipson EJ, Ascierto PA, Matamala L, Castillo Gutiérrez E, et al. Relatlimab and nivolumab versus nivolumab in untreated advanced melanoma. N Engl J Med. 2022;386(1):24–34. 34986285 10.1056/NEJMoa2109970 PMC9844513

[111] Li Y, Ju M, Miao Y, Zhao L, Xing L, Wei M. Advancement of anti-LAG ‐3 in cancer therapy. FASEB J. 2023;37(11):e23236. 37846808 10.1096/fj.202301018R

[112] Qin S, Xu L, Yi M, Yu S, Wu K, Luo S. Novel immune checkpoint targets: moving beyond PD-1 and CTLA-4. Mol Cancer. 2019;18(1):155. 31690319 10.1186/s12943-019-1091-2 PMC6833286

[113] Wolf Y, Anderson AC, Kuchroo VK. TIM3 comes of age as an inhibitory receptor. Nat Rev Immunol. 2020;20(3):173–85. 31676858 10.1038/s41577-019-0224-6 PMC7327798

[114] Sakuishi K, Apetoh L, Sullivan JM, Blazar BR, Kuchroo VK, Anderson AC. Targeting Tim-3 and PD-1 pathways to reverse T cell exhaustion and restore anti-tumor immunity. J Exp Med. 2010;207(10):2187–94. 20819927 10.1084/jem.20100643 PMC2947065

[115] Koyama S, Akbay EA, Li YY, Herter-Sprie GS, Buczkowski KA, Richards WG, et al. Adaptive resistance to therapeutic PD-1 Blockade is associated with upregulation of alternative immune checkpoints. Nat Commun. 2016;7:10501. 26883990 10.1038/ncomms10501 PMC4757784

[116] Ngiow SF, von Scheidt B, Akiba H, Yagita H, Teng MWL, Smyth MJ. Anti-TIM3 antibody promotes T cell IFN-γ–Mediated antitumor immunity and suppresses established tumors. Cancer Res. 2011;71(10):3540–51. 21430066 10.1158/0008-5472.CAN-11-0096

[117] Fourcade J, Sun Z, Pagliano O, Guillaume P, Luescher IF, Sander C, et al. CD8(+) T cells specific for tumor antigens can be rendered dysfunctional by the tumor microenvironment through upregulation of the inhibitory receptors BTLA and PD-1. Cancer Res. 2012;72(4):887–96. 22205715 10.1158/0008-5472.CAN-11-2637 PMC3288235

[118] Ziogas DC, Theocharopoulos C, Lialios PP, Foteinou D, Koumprentziotis IA, Xynos G, et al. Beyond CTLA-4 and PD-1 inhibition: novel immune checkpoint molecules for melanoma treatment. Cancers. 2023;15(10):2718. 37345056 10.3390/cancers15102718 PMC10216291

[119] Kuklinski LF, Yan S, Li Z, Fisher JL, Cheng C, Noelle RJ, et al. VISTA expression on tumor-infiltrating inflammatory cells in primary cutaneous melanoma correlates with poor disease-specific survival. Cancer Immunol Immunother. 2018;67(7):1113–21. 29737375 10.1007/s00262-018-2169-1 PMC11028124

[120] Powderly J, Patel MR, Lee JJ, Brody J, Meric-Bernstam F, Hamilton E, et al. CA-170, a first in class oral small molecule dual inhibitor of immune checkpoints PD-L1 and VISTA, demonstrates tumor growth Inhibition in pre-clinical models and promotes T cell activation in phase 1 study. Ann Oncol. 2017;28:v405–6.

[121] June CH, O’Connor RS, Kawalekar OU, Ghassemi S, Milone MC. CAR T cell immunotherapy for human cancer. Science. 2018;359(6382):1361–5. 29567707 10.1126/science.aar6711

[122] Adoptive cell transfer therapy [Internet]. Melanoma Research Alliance. [cited 2024 May 10]. Available from: https://www.curemelanoma.org/patient-eng/melanoma-treatment/therapies-in-development/adoptive-cell-transfer-therapy#panel-clinical-trial-locator

[123] Morgan DA, Ruscetti FW, Gallo R. Selective in vitro growth of T lymphocytes from normal human bone marrows. Science. 1976;193(4257):1007–8. 181845 10.1126/science.181845

[124] Rosenberg SA, Lotze MT, Muul LM, Leitman S, Chang AE, Ettinghausen SE, et al. Observations on the systemic administration of autologous lymphokine-activated killer cells and Recombinant interleukin-2 to patients with metastatic cancer. N Engl J Med. 1985;313(23):1485–92. 3903508 10.1056/NEJM198512053132327

[125] Muul LM, Spiess PJ, Director EP, Rosenberg SA. Identification of specific cytolytic immune responses against autologous tumor in humans bearing malignant melanoma. J Immunol Baltim Md 1950. 1987;138(3):989–95. 3100623

[126] Rosenberg SA, Packard BS, Aebersold PM, Solomon D, Topalian SL, Toy ST, et al. Use of tumor-infiltrating lymphocytes and interleukin-2 in the immunotherapy of patients with metastatic melanoma. A preliminary report. N Engl J Med. 1988;319(25):1676–80. 3264384 10.1056/NEJM198812223192527

[127] Dafni U, Michielin O, Lluesma SM, Tsourti Z, Polydoropoulou V, Karlis D, et al. Efficacy of adoptive therapy with tumor-infiltrating lymphocytes and Recombinant interleukin-2 in advanced cutaneous melanoma: a systematic review and meta-analysis. Ann Oncol. 2019;30(12):1902–13. 31566658 10.1093/annonc/mdz398

[128] Rosenberg SA. Lymphocytes as a living drug for cancer. Science. 2024;385(6704):25–6. 38963837 10.1126/science.adp1130

[129] Dudley ME, Wunderlich JR, Robbins PF, Yang JC, Hwu P, Schwartzentruber DJ, et al. Cancer regression and autoimmunity in patients after clonal repopulation with antitumor lymphocytes. Science. 2002;298(5594):850–4. 12242449 10.1126/science.1076514 PMC1764179

[130] Keam SJ, Lifileucel. First approval. Mol Diagn Ther. 2024;28(3):339–44. 38625642 10.1007/s40291-024-00708-y

[131] Klobuch S, Seijkens TTP, Schumacher TN, Haanen JBAG. Tumour-infiltrating lymphocyte therapy for patients with advanced-stage melanoma. Nat Rev Clin Oncol [Internet]. 2024;[cited 2024 Aug 6]; Available from: https://www.nature.com/articles/s41571-023-00848-w 10.1038/s41571-023-00848-w 38191921

[132] Center for Drug Evaluation and Research. FDA grants accelerated approval to lifileucel for unresectable or metastatic melanoma, U.S. Food and Drug Administration. Available at: https://www.fda.gov/drugs/resources-information-approved-drugs/fda-grants-accelerated-approval-lifileucel-unresectable-or-metastatic-melanoma (Accessed: 06 August 2024).

[133] Chesney J, Lewis KD, Kluger H, Hamid O, Whitman E, Thomas S, et al. Efficacy and safety of lifileucel, a one-time autologous tumor-infiltrating lymphocyte (TIL) cell therapy, in patients with advanced melanoma after progression on immune checkpoint inhibitors and targeted therapies: pooled analysis of consecutive cohorts of the C-144-01 study. J Immunother Cancer. 2022;10(12):e005755. 36600653 10.1136/jitc-2022-005755 PMC9748991

[134] Rohaan MW, Borch TH, Van Den Berg JH, Met Ö, Kessels R, Geukes Foppen MH, et al. Tumor-Infiltrating lymphocyte therapy or ipilimumab in advanced melanoma. N Engl J Med. 2022;387(23):2113–25. 36477031 10.1056/NEJMoa2210233

[135] Mullinax JE, Hall M, Prabhakaran S, Weber J, Khushalani N, Eroglu Z, et al. Combination of ipilimumab and adoptive cell therapy with Tumor-Infiltrating lymphocytes for patients with metastatic melanoma. Front Oncol. 2018;8:44. 29552542 10.3389/fonc.2018.00044 PMC5840208

[136] Hall MS, Mullinax JE, Cox CA, Hall AM, Beatty MS, Blauvelt J, et al. Combination Nivolumab, CD137 Agonism, and adoptive cell therapy with Tumor-Infiltrating lymphocytes for patients with metastatic melanoma. Clin Cancer Res. 2022;28(24):5317–29. 36215121 10.1158/1078-0432.CCR-22-2103 PMC10324027

[137] Seitter SJ, Sherry RM, Yang JC, Robbins PF, Shindorf ML, Copeland AR, et al. Impact of prior treatment on the efficacy of adoptive transfer of Tumor-Infiltrating lymphocytes in patients with metastatic melanoma. Clin Cancer Res Off J Am Assoc Cancer Res. 2021;27(19):5289–98. 10.1158/1078-0432.CCR-21-1171 PMC8857302 34413159

[138] Kerr MD, McBride DA, Chumber AK, Shah NJ. Combining therapeutic vaccines with chemo- and immunotherapies in the treatment of cancer. Expert Opin Drug Discov. 2021;16(1):89–99. 32867561 10.1080/17460441.2020.1811673 PMC7785654

[139] Bidram M, Zhao Y, Shebardina NG, Baldin AV, Bazhin AV, Ganjalikhany MR, et al. mRNA-Based cancer vaccines: A therapeutic strategy for the treatment of melanoma patients. Vaccines. 2021;9(10):1060. 34696168 10.3390/vaccines9101060 PMC8540049

[140] Kaczmarek M, Poznańska J, Fechner F, Michalska N, Paszkowska S, Napierała A, et al. Cancer Vaccine Therapeutics: Limitations Effectiveness—A Literature Rev Cells. 2023;12(17):2159. 10.3390/cells12172159 PMC10486481 37681891

[141] Samarasekera U. New partnership to boost UK cancer vaccine research. Lancet Oncol. 2023;24(2):132. 36642081 10.1016/S1470-2045(23)00011-6 PMC9836399

[142] Khattak A, Weber JS, Meniawy T, Taylor MH, Ansstas G, Kim KB, et al. Distant metastasis-free survival results from the randomized, phase 2 mRNA-4157-P201/KEYNOTE-942 trial. J Clin Oncol. 2023;41(17suppl):LBA9503–9503.

[143] Weber JS, Carlino MS, Khattak A, Meniawy T, Ansstas G, Taylor MH, et al. Individualised neoantigen therapy mRNA-4157 (V940) plus pembrolizumab versus pembrolizumab monotherapy in resected melanoma (KEYNOTE-942): a randomised, phase 2b study. Lancet Lond Engl. 2024;403(10427):632–44. 10.1016/S0140-6736(23)02268-7 38246194

[144] Oladejo M, Paulishak W, Wood L. Synergistic potential of immune checkpoint inhibitors and therapeutic cancer vaccines. Semin Cancer Biol. 2023;88:81–95. 36526110 10.1016/j.semcancer.2022.12.003

[145] Jhawar SR, Thandoni A, Bommareddy PK, Hassan S, Kohlhapp FJ, Goyal S, et al. Oncolytic Viruses-Natural and genetically engineered cancer immunotherapies. Front Oncol. 2017;7:202. 28955655 10.3389/fonc.2017.00202 PMC5600978

[146] Ferrucci PF, Pala L, Conforti F, Cocorocchio E. Talimogene Laherparepvec (T-VEC): an intralesional cancer immunotherapy for advanced melanoma. Cancers. 2021;13(6):1383. 33803762 10.3390/cancers13061383 PMC8003308

[147] Andtbacka RHI, Collichio F, Harrington KJ, Middleton MR, Downey G, Ӧhrling K, et al. Final analyses of optim: a randomized phase III trial of talimogene Laherparepvec versus granulocyte-macrophage colony-stimulating factor in unresectable stage III–IV melanoma. J Immunother Cancer. 2019;7(1):145. 31171039 10.1186/s40425-019-0623-z PMC6554874

[148] Robinson C, Xu MM, Nair SK, Beasley GM, Rhodin KE. Oncolytic viruses in melanoma. Front Biosci Landmark Ed. 2022;27(2):63. 35227006 10.31083/j.fbl2702063 PMC9888358

[149] Thomas S, Kuncheria L, Roulstone V, Kyula JN, Mansfield D, Bommareddy PK, et al. Development of a new fusion-enhanced oncolytic immunotherapy platform based on herpes simplex virus type 1. J Immunother Cancer. 2019;7(1):214. 31399043 10.1186/s40425-019-0682-1 PMC6689178

[150] Wong MK, Milhem MM, Sacco JJ, Michels J, In GK, Muñoz Couselo E, et al. RP1 combined with nivolumab in advanced Anti–PD-1–Failed melanoma (IGNYTE). J Clin Oncol. 2025;0(0):JCO–25. 10.1200/JCO-25-01346 PMC12622257 40627813

[151] Anticancer immunotherapy by CTLA-. 4 blockade relies on the gut microbiota | Science [Internet]. [cited 2021 Apr 27]. Available from: https://science.sciencemag.org/content/350/6264/1079 10.1126/science.aad1329 PMC4721659 26541610

[152] Routy B, Chatelier EL, Derosa L, Duong CPM, Alou MT, Daillère R, et al. Gut Microbiome influences efficacy of PD-1–based immunotherapy against epithelial tumors. Science. 2018;359(6371):91–7. 29097494 10.1126/science.aan3706

[153] Yang M, Wang Y, Yuan M, Tao M, Kong C, Li H, et al. Antibiotic administration shortly before or after immunotherapy initiation is correlated with poor prognosis in solid cancer patients: an up-to-date systematic review and meta-analysis. Int Immunopharmacol. 2020;88:106876. 32799113 10.1016/j.intimp.2020.106876

[154] Huang XZ, Gao P, Song YX, Xu Y, Sun JX, Chen XW et al. Antibiotic use and the efficacy of immune checkpoint inhibitors in cancer patients: a pooled analysis of 2740 cancer patients. Oncoimmunology [Internet]. 2019;[cited 2020 Nov 29];8(12). Available from: https://www.ncbi.nlm.nih.gov/pmc/articles/PMC6844307/10.1080/2162402X.2019.1665973 PMC6844307 31741763

[155] Morganti S, Curigliano G. Combinations using checkpoint Blockade to overcome resistance. Ecancermedicalscience. 2020;14:1148. 33574893 10.3332/ecancer.2020.1148 PMC7864692

[156] Haibe Y, El Husseini Z, El Sayed R, Shamseddine A. Resisting Resistance to Immune Checkpoint Therapy: A Systematic Review. Int J Mol Sci [Internet]. 2020;[cited 2021 Apr 27];21(17). Available from: https://www.ncbi.nlm.nih.gov/pmc/articles/PMC7504220/10.3390/ijms21176176 PMC7504220 32867025

[157] Evans S, Martini D, Magod B, Olsen T, Brown J, Yantorni L et al. 255 Efficacy of sequential immune checkpoint inhibition (ICI) in patients with genitourinary malignancies. J Immunother Cancer [Internet]. 2020;[cited 2021 Apr 27];8(Suppl 3). Available from: https://jitc.bmj.com/content/8/Suppl_3/A154

